# Supplementary material for: Atomic resolution structures of the methane-activating enzyme in anaerobic methanotrophy reveal extensive post-translational modifications
Source: Nat Commun. 2025 Sep 5;16:8229. doi: 10.1038/s41467-025-63387-1 (PMC12413448; doi:10.1038/s41467-025-63387-1)
Supplement: Supplementary file 1 — Supplementary Information [file 41467_2025_63387_MOESM1_ESM.pdf]

Atomic resolution structures of the methane-activating enzyme in anaerobic methanotrophy reveal extensive post-translational modifications

Marie-C. Müller, Martijn Wissink, Priyadarshini Mukherjee, Nicole Von Possel, Rafael Laso-Pérez, Sylvain Engilberge, Philippe Carpentier, Jörg Kahnt, Gunter Wegener, Cornelia U. Welte, Tristan Wagner.

Supplementary Information.

This file contains the Supplementary Figures S1 to S23 and Supplementary references.

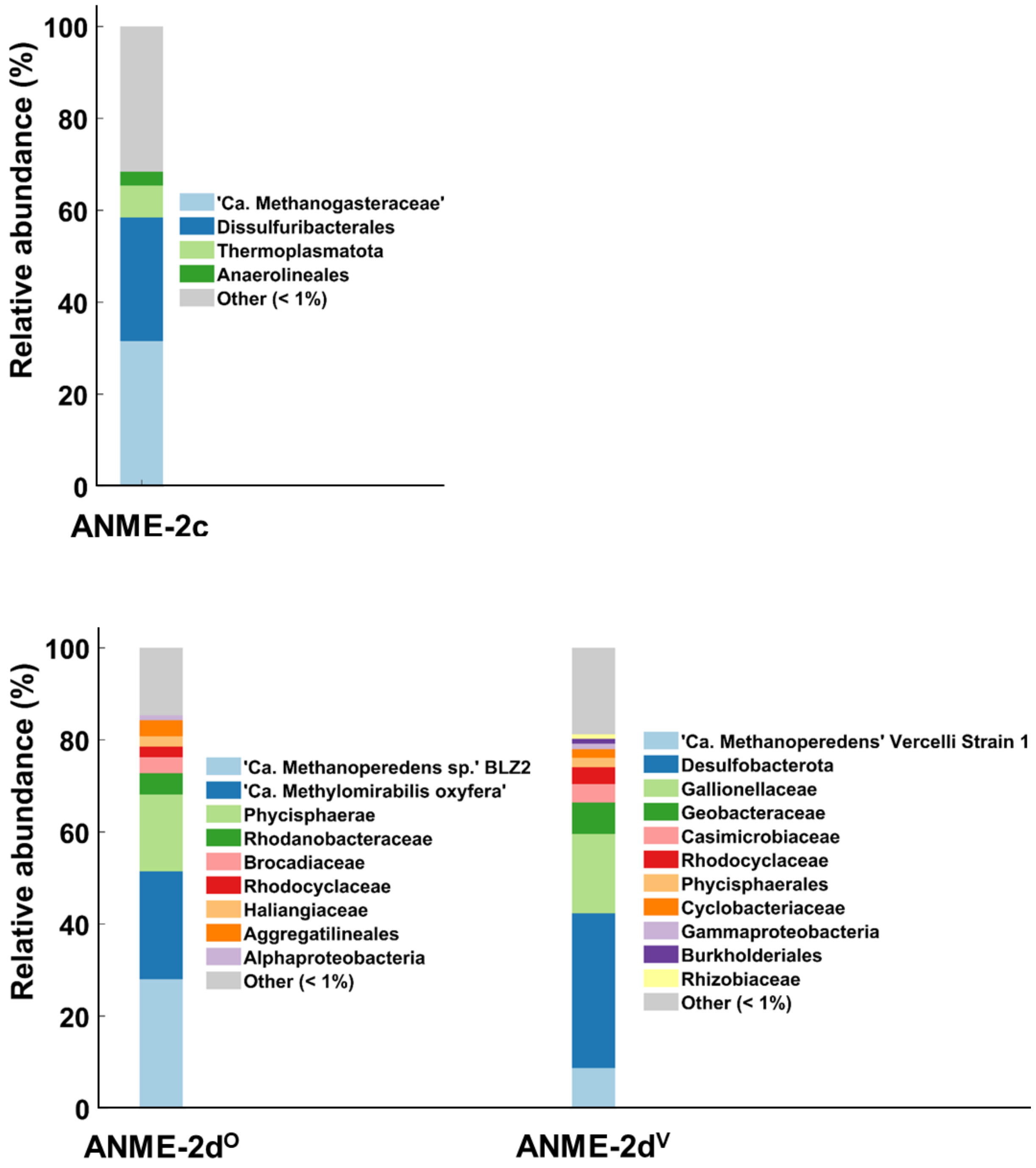

Supplementary Figure S1. Metagenomic read-based relative abundances of ANME enrichments. SingleM was used for taxonomical read-classification on three ANME enrichments using the GTDB-Tk database version 2.4.0 with raw reads as input(1).

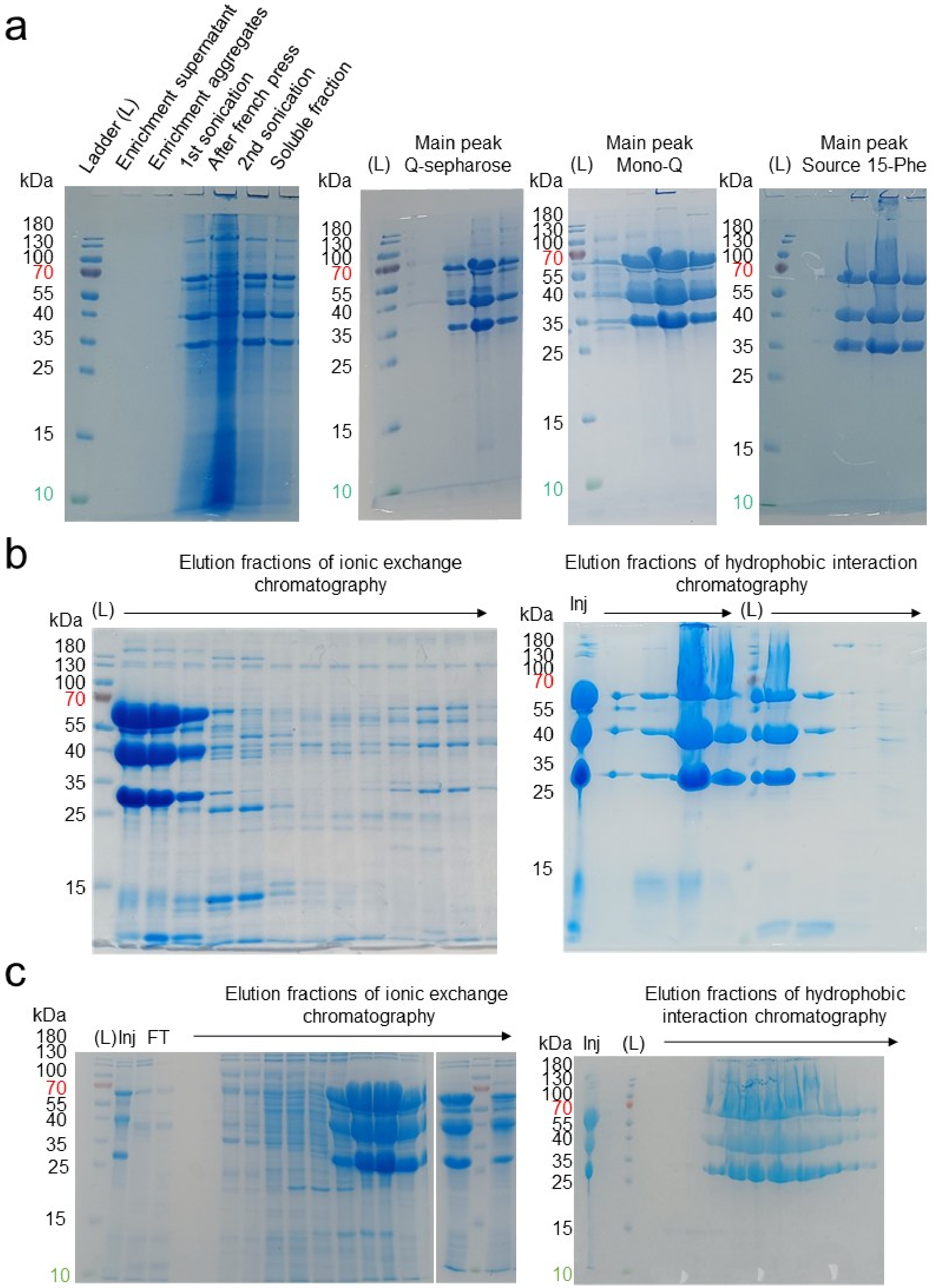

Supplementary Figure S2. Continued on the next page.

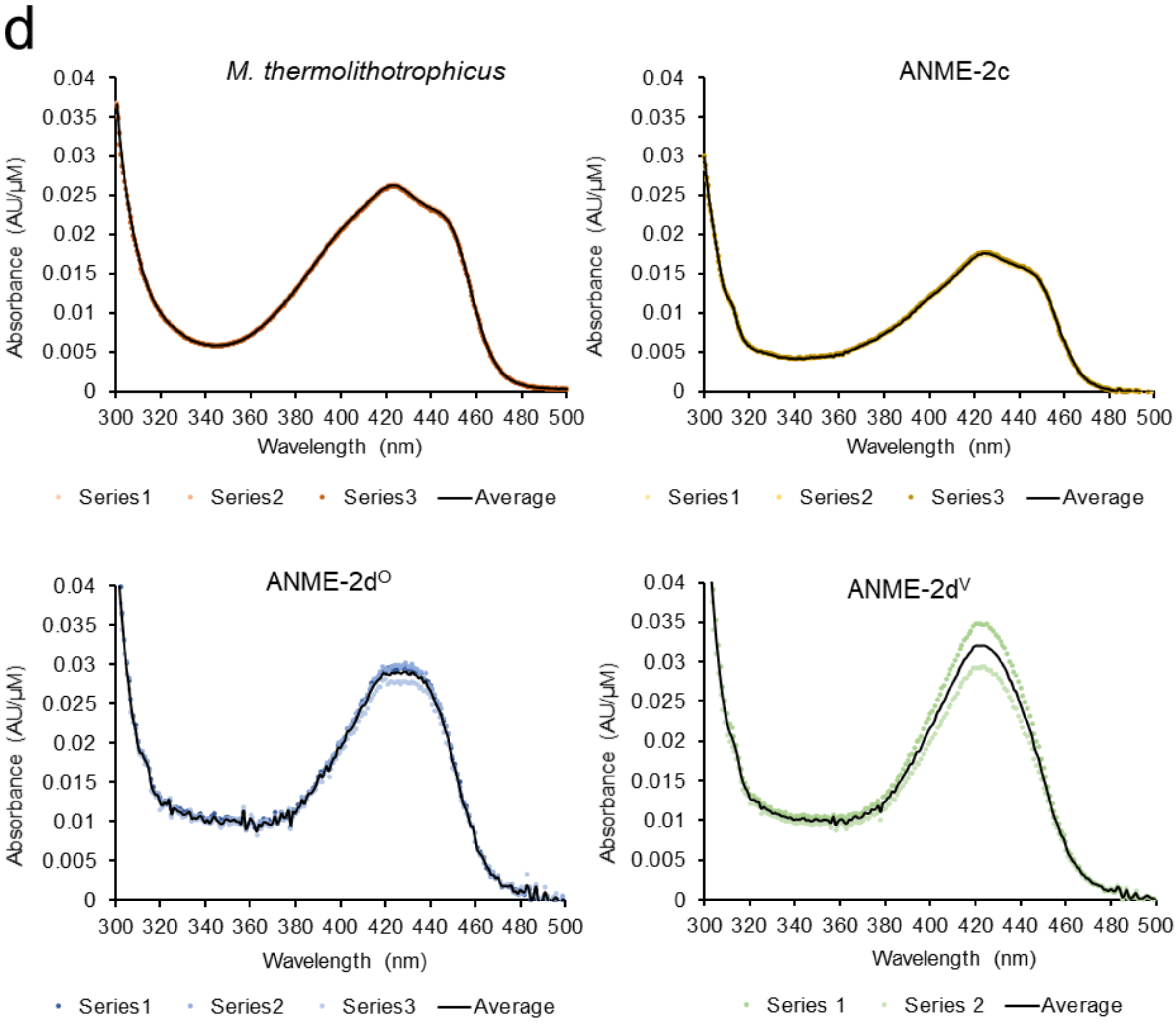

Supplementary Figure S2. Purification profiles of ANME-2 MCRs and their UV/Visible spectra. (**a-c**) SDS-PAGE of different purification steps. L, Inj, and FT stand for Ladder, injected sample on the chromatography column, and flow through, respectively. Panels a, b, and c correspond to the MCR purification from ANME-2c, ANME-2d^{O}, and ANME-2d^{V}, respectively. The purification of MCR from ANME-2c and ANME-2d^{V} has been performed once, and the purification of MCR from ANME-2d^{O} has been performed three times. (**d**) UV-visible spectrum of ANME-2 MCRs compared to methanogenic homolog from *Methanothermococcus thermolithotrophicus*(2). The measured signal is in absorbance (AU) per µM of measured MCR. Measurements have been performed in technical triplicate, except for MCR ANME-2d^{V}, which has been measured in technical duplicate.

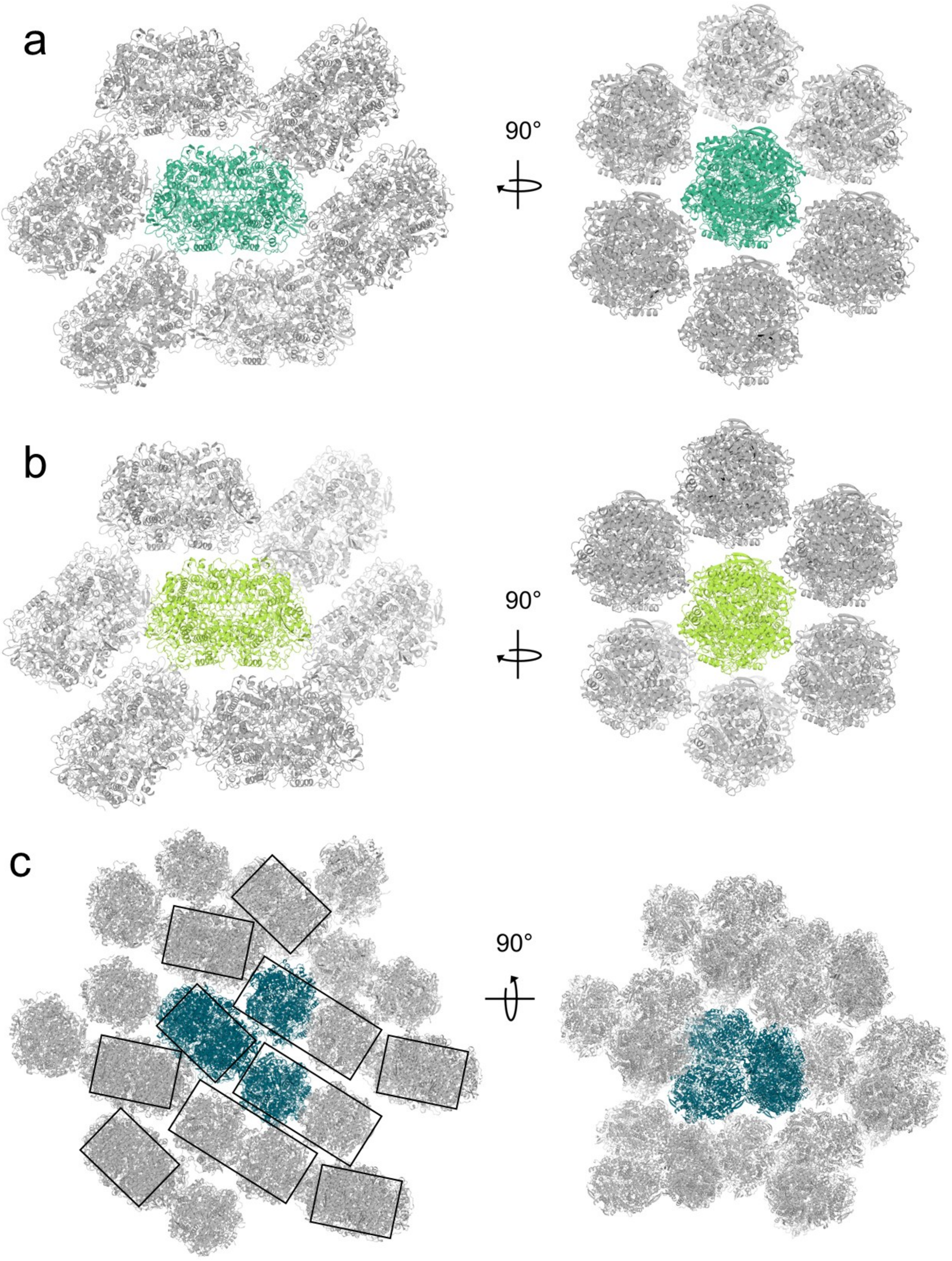

Supplementary Figure S3. Presentation of ANME-2 MCR crystalline packing. Packing of MCR from ANME-2d^{O} (**a**), ANME-2d^{V} (**b**), and ANME-2c (**c**). MCR units are shown in cartoons. The asymmetric unit content is coloured, while the symmetry mates are grey. Black boxes indicate observable dimeric units for ANME-2c MCR, which might come from a crystallisation artefact.

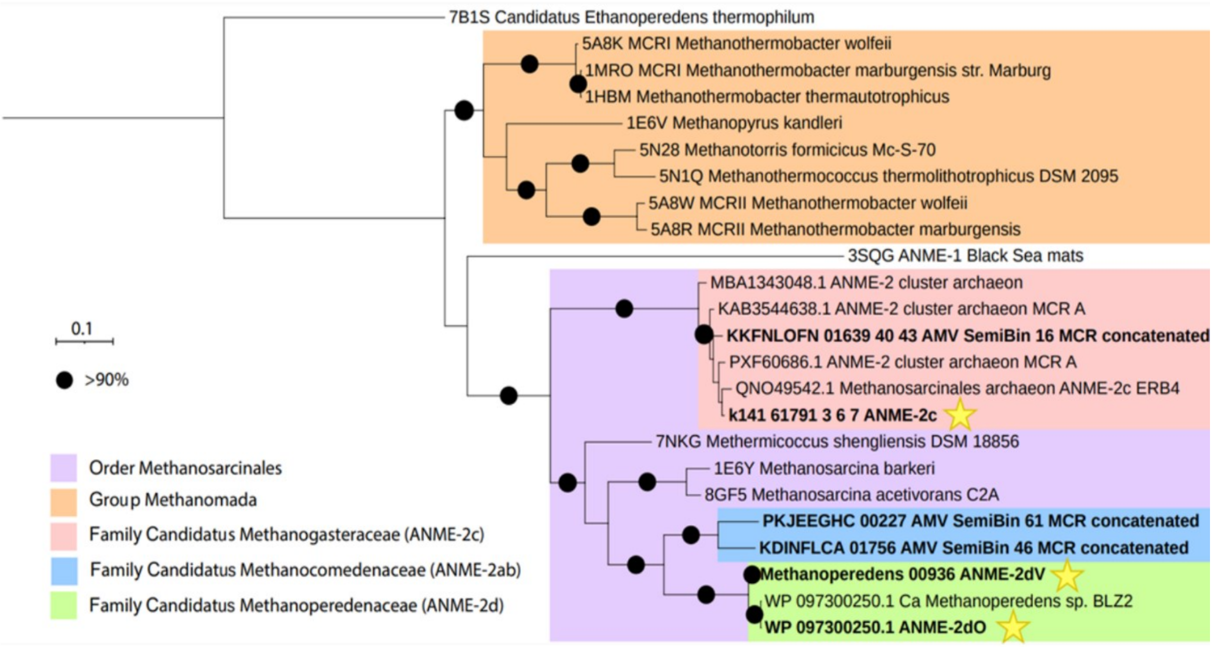

Supplementary Figure S4. Phylogeny tree of the structurally characterised MCRs and close sequence homologues from the marine ANME-2 MCR. The tree represents the concatenation of the α, β, and γ MCR subunits. The models studied in this work are highlighted with yellow stars.

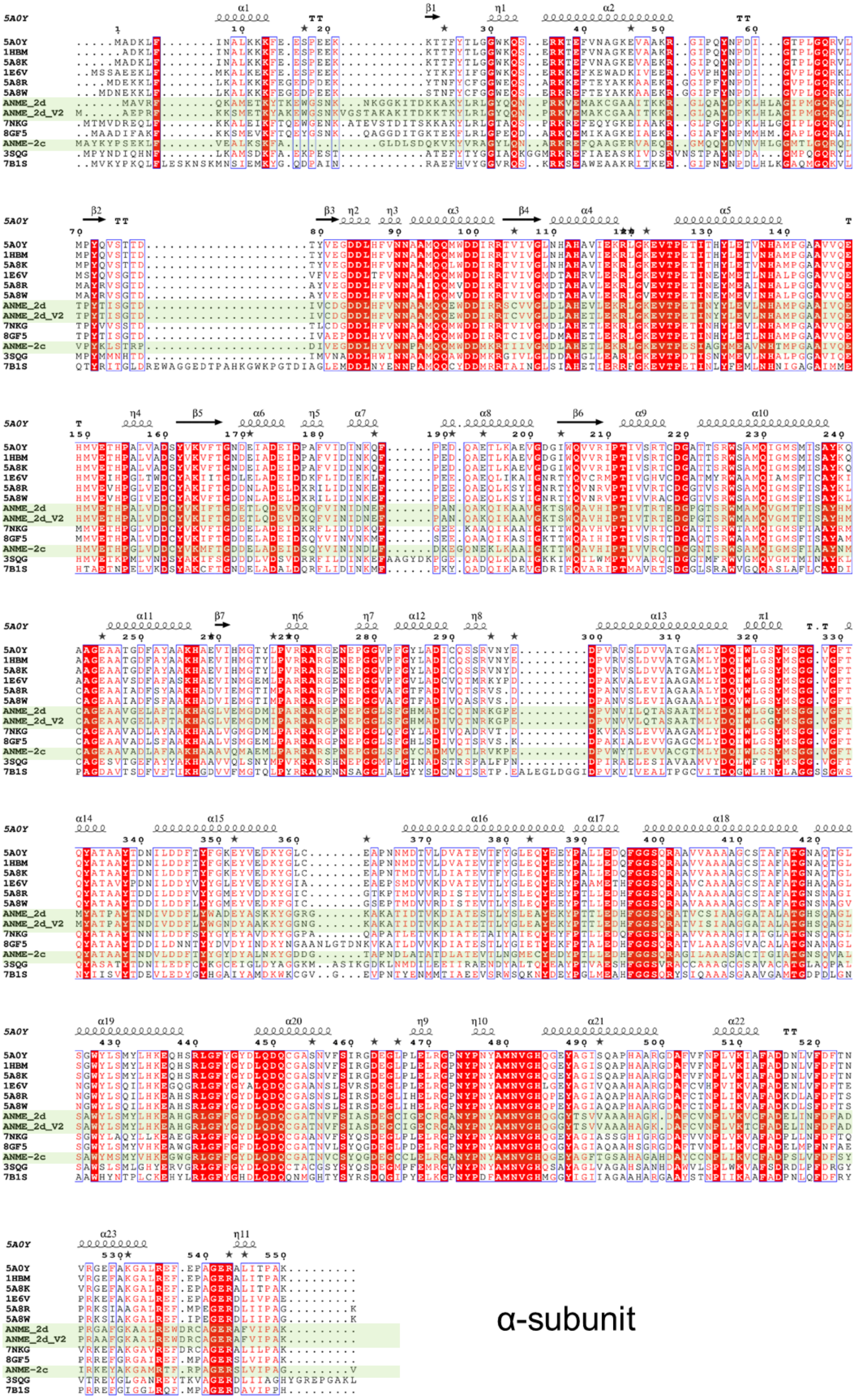
α-subunit

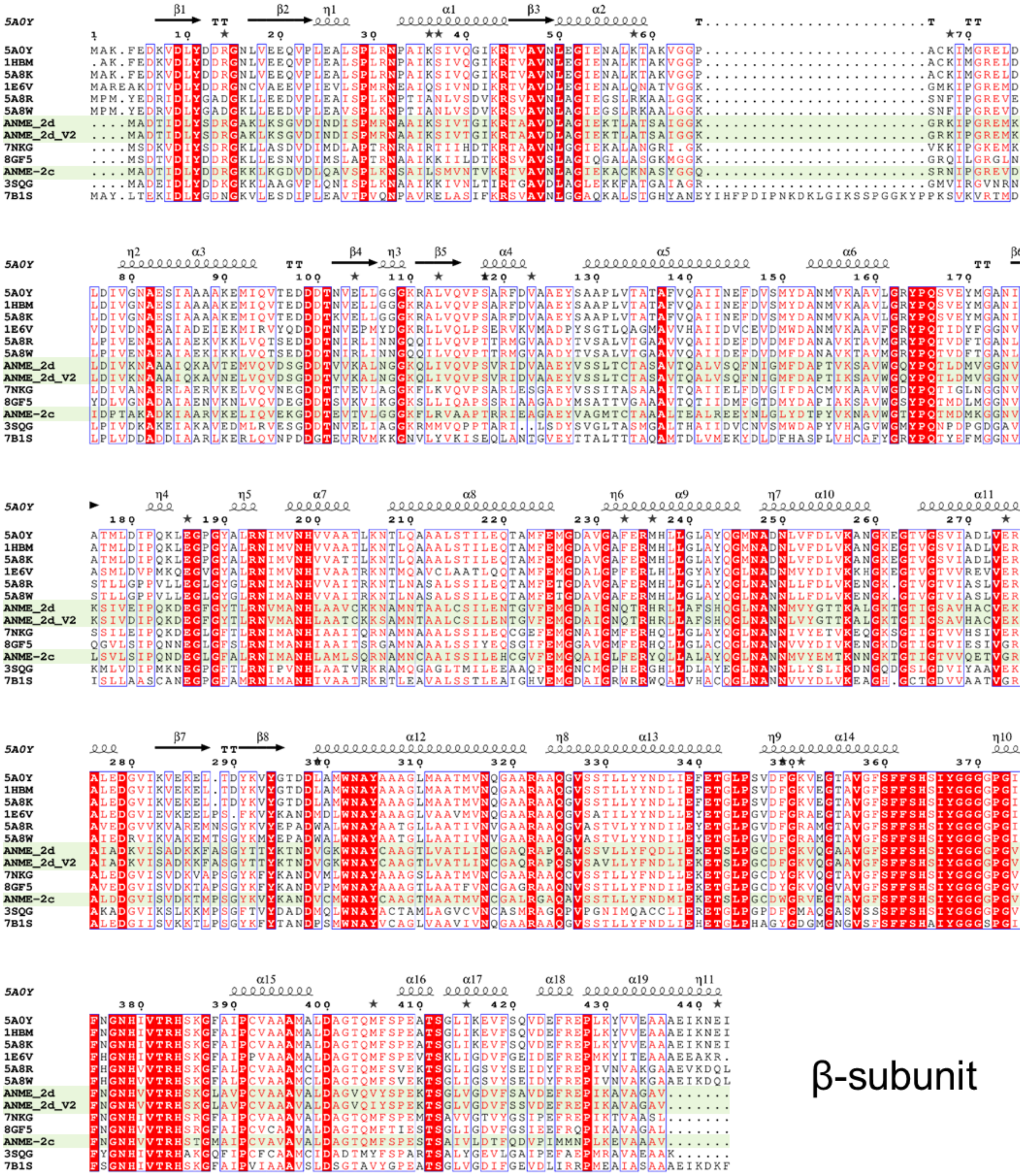
5A0Y
1HBM
5A8K
1E6V
5A8R
5A8W
ANME_2d
ANME_2d_V2
7NKG
8GF5
ANME-2c
3SQG
7B1S
β-subunit

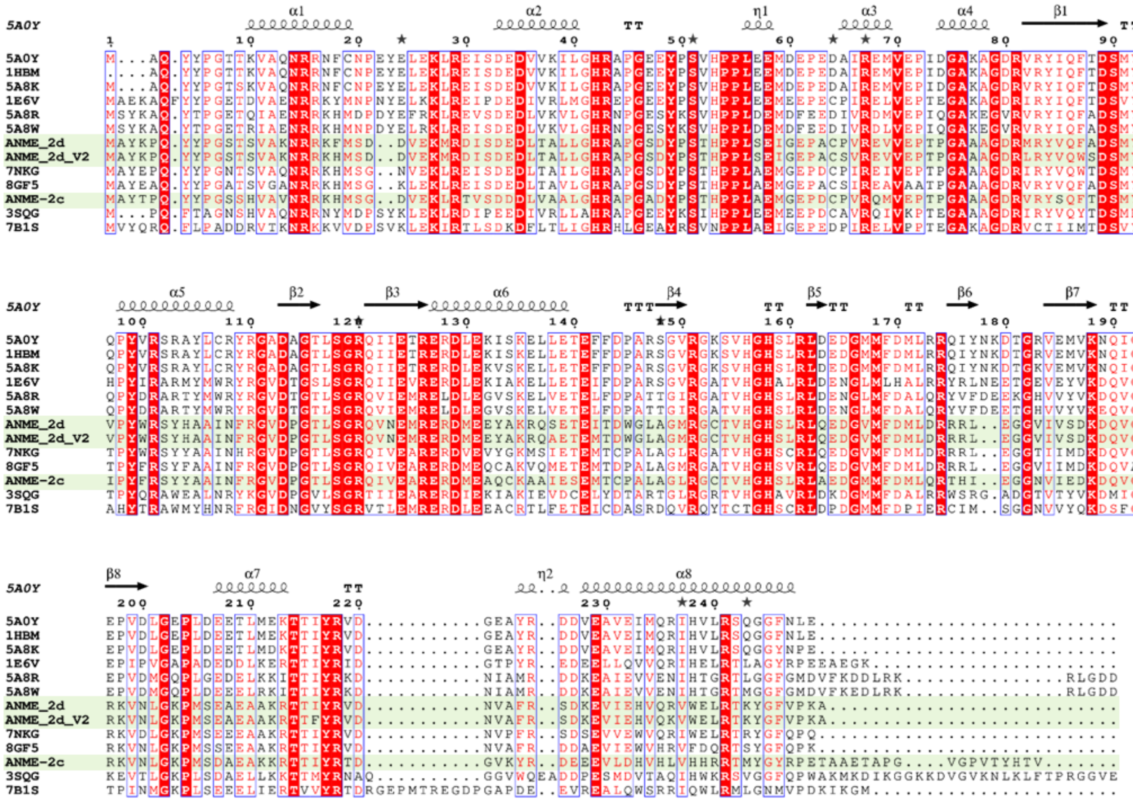

γ-subunit

Supplementary Figure S5. Sequence alignment of the structurally characterised MCR homologs. ESPript alignment(3) of marine ANME-2c, freshwater ANME-2d^{O} (ANME_2d), freshwater ANME-2d^{V} (ANME_2d_V2) and structurally characterised homologs from *Methanothermobacter marburgensis* isoform I (PDB code: 5A0Y), *Methanothermobacter thermautotrophicus* (1HBM), *Methanothermobacter wolfeii* isoform I (5A8K), *Methanopyrus kandleri* (1E6V), *Methanothermobacter marburgensis* isoform II (5A8R), *Methanothermobacter wolfeii* isoform II (5A8W), *Methermicoccus shengliensis* (7NKG), *Methanosarcina acetivorans* (8GF5), ANME-1 from Black Sea mats (3SQG) and '*Candidatus* Ethanoperedens thermophilum' (7B1S). ANME-2 MCR sequences are highlighted in green.

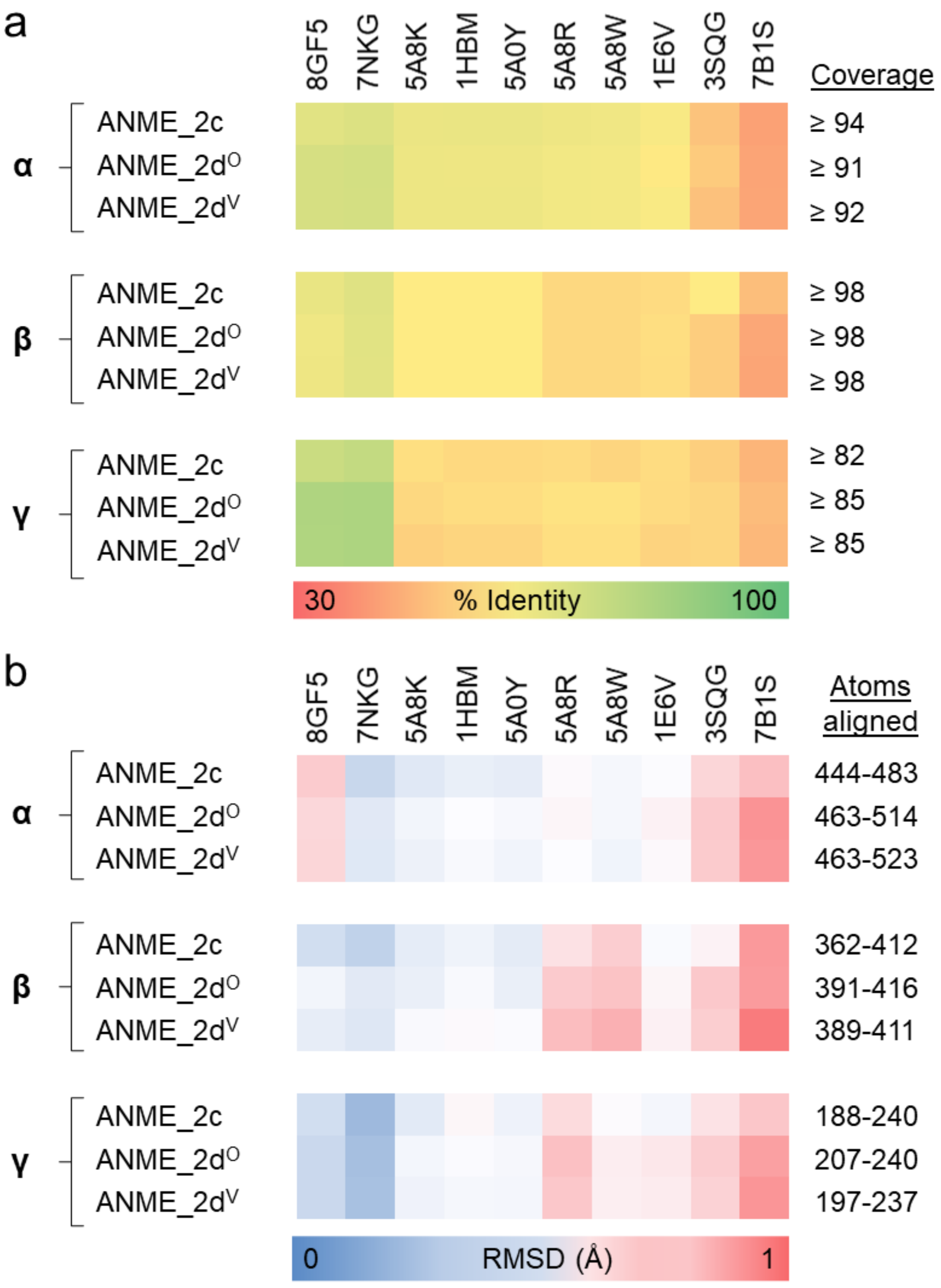

Supplementary Figure S6. Sequence and structural similarity of characterised MCR homologs. (**a**) Sequence similarity of the α-, β- and γ-subunits of ANME-2 MCRs to structural homologs. The percentage of identity is displayed as a gradient from red to green, ranging from 30% to 100% sequence identity. The coverage for all samples is summarised on the right. (**b**) Structural similarity of the α-, β- and γ-subunits of ANME-2 MCRs to structural homologs. The RMSD is displayed as a gradient from blue to red, ranging from 0 to 1 Å. The aligned Cα atoms for all samples are summarised on the right. Listed homologs are *M. marburgensis* isoform I (PDB code: 5A0Y), *M. thermautotrophicus* (1HBM), *M. wolfeii* isoform I (5A8K), *M. kandleri* (1E6V), *M. marburgensis* isoform II (5A8R), *M. wolfeii* isoform II (5A8W), *M. shengliensis* (7NKG), *M. acetivorans* (8GF5), ANME-1 (3SQG), and '*Ca*. E. thermophilum' (7B1S).

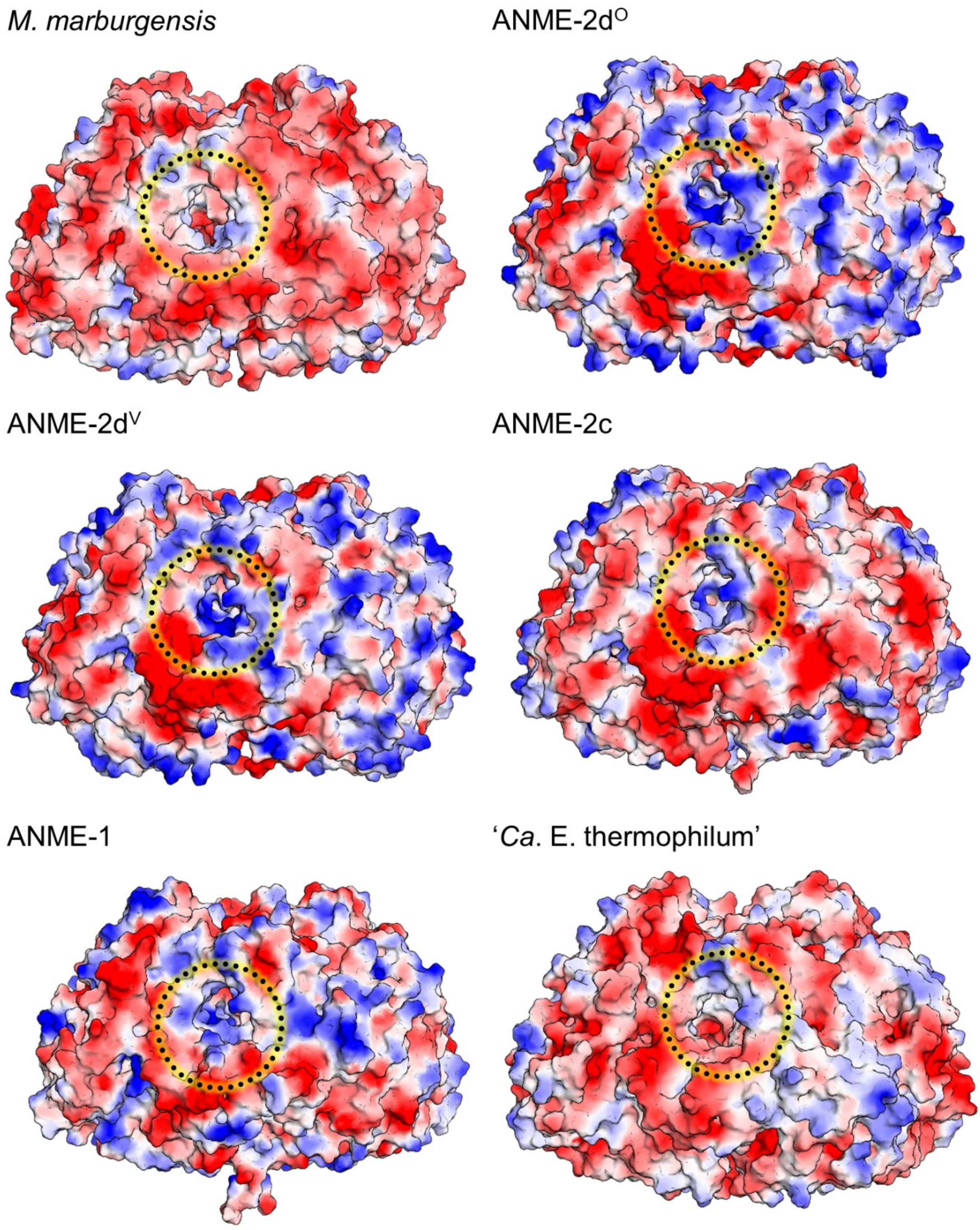

Supplementary Figure S7. Electrostatic potential on the surface of MCRs. The vacuum electrostatic potential of MCR is shown as a gradient from red to blue for a more negative to a positive potential, respectively. Dashed circles with a yellow glow highlight the active site entrance. PDB codes for the models are the following: *M. marburgensis* isoform I (PDB code: 5A0Y), ANME-1 (3SQG), and ‘*Ca*. E. thermophilum’ (7B1S).

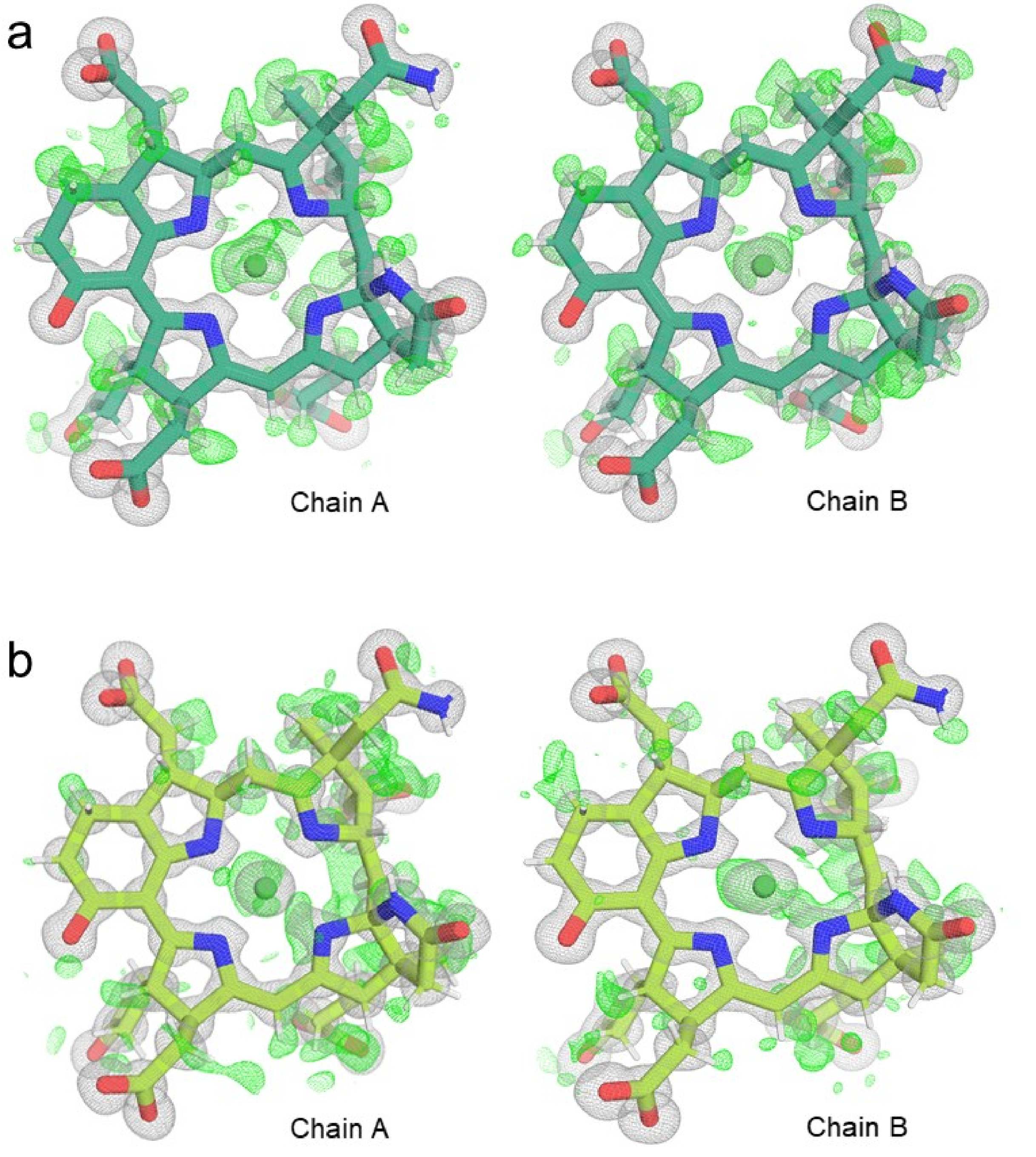

Supplementary Figure S8. Electron density map of F_{430}. The electron density is shown for (**a**) ANME-2d^{O} MCR and (**b**) ANME-2d^{V} MCR as grey mesh for the $2F_o–F_c$ map at 1 σ and green mesh for the $F_o–F_c$ map at 2.8 σ. F_{430} is shown as sticks. Atoms are coloured in red for O, blue for N, green for Ni, and white for riding H. The map was generated with non-hydrogenated F_{430}s.

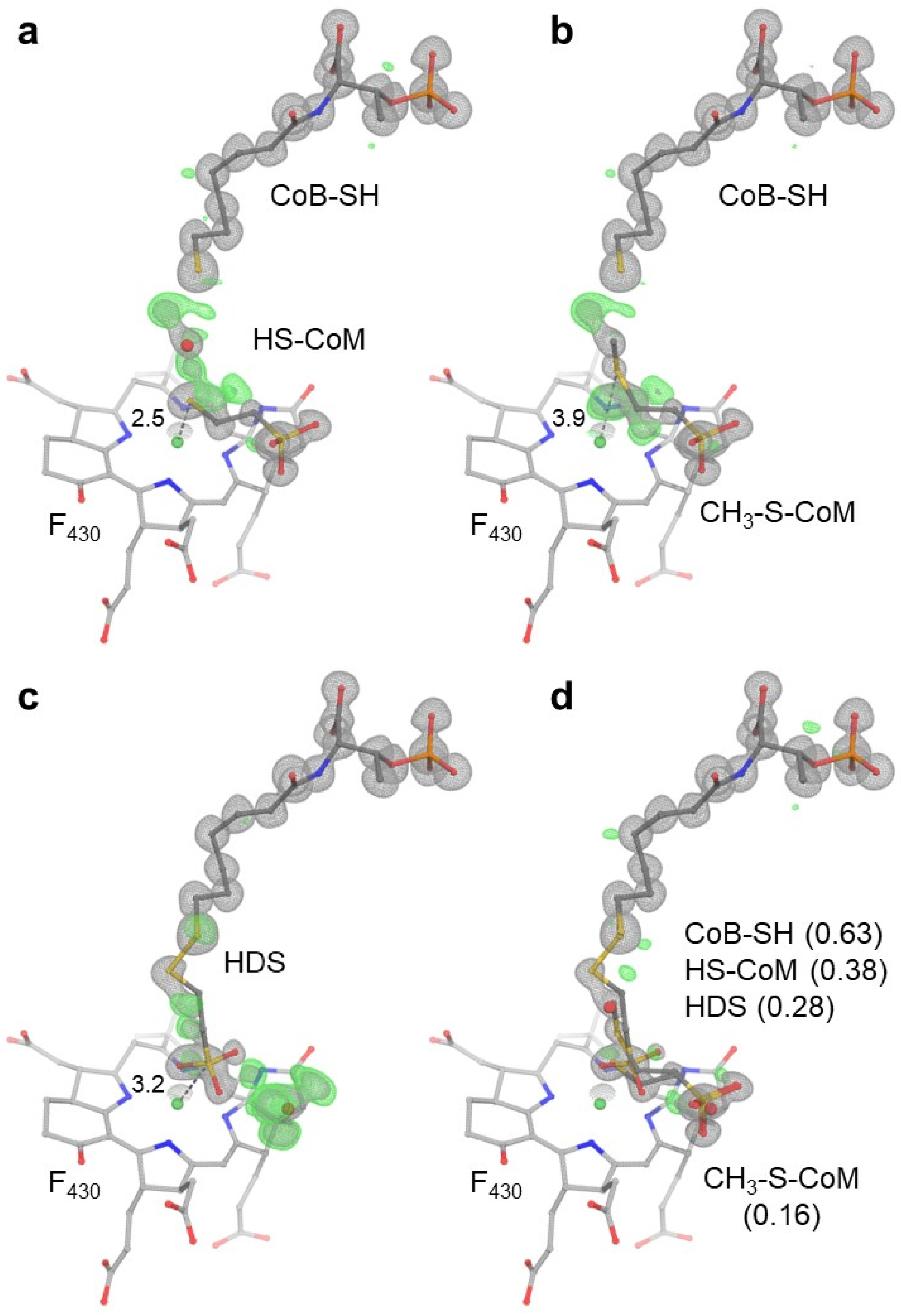

Supplementary Figure S9. Ligand modelling in ANME-2d^{V} MCR active site. Ligand mixtures were modelled as follows: (**a**) Coenzyme B and coenzyme M, (**b**) coenzyme B and methyl-coenzyme M, (**c**) heterodisulfide of coenzyme M and coenzyme B and (**d**) all four ligands. Models were refined with ligands at 1.0 occupancy except for (**d**), where partial occupancies are indicated in brackets. Ligands are shown as sticks with atoms coloured in red for O, blue for N, yellow for S, green for Ni and orange for P. The $2F_o{-}F_c$ map is contoured at 1 σ and the $F_o{-}F_c$ map is contoured at 4 σ and shown as grey and green mesh, respectively. The distance (Å) between the S and Ni atom is shown as black dashes.

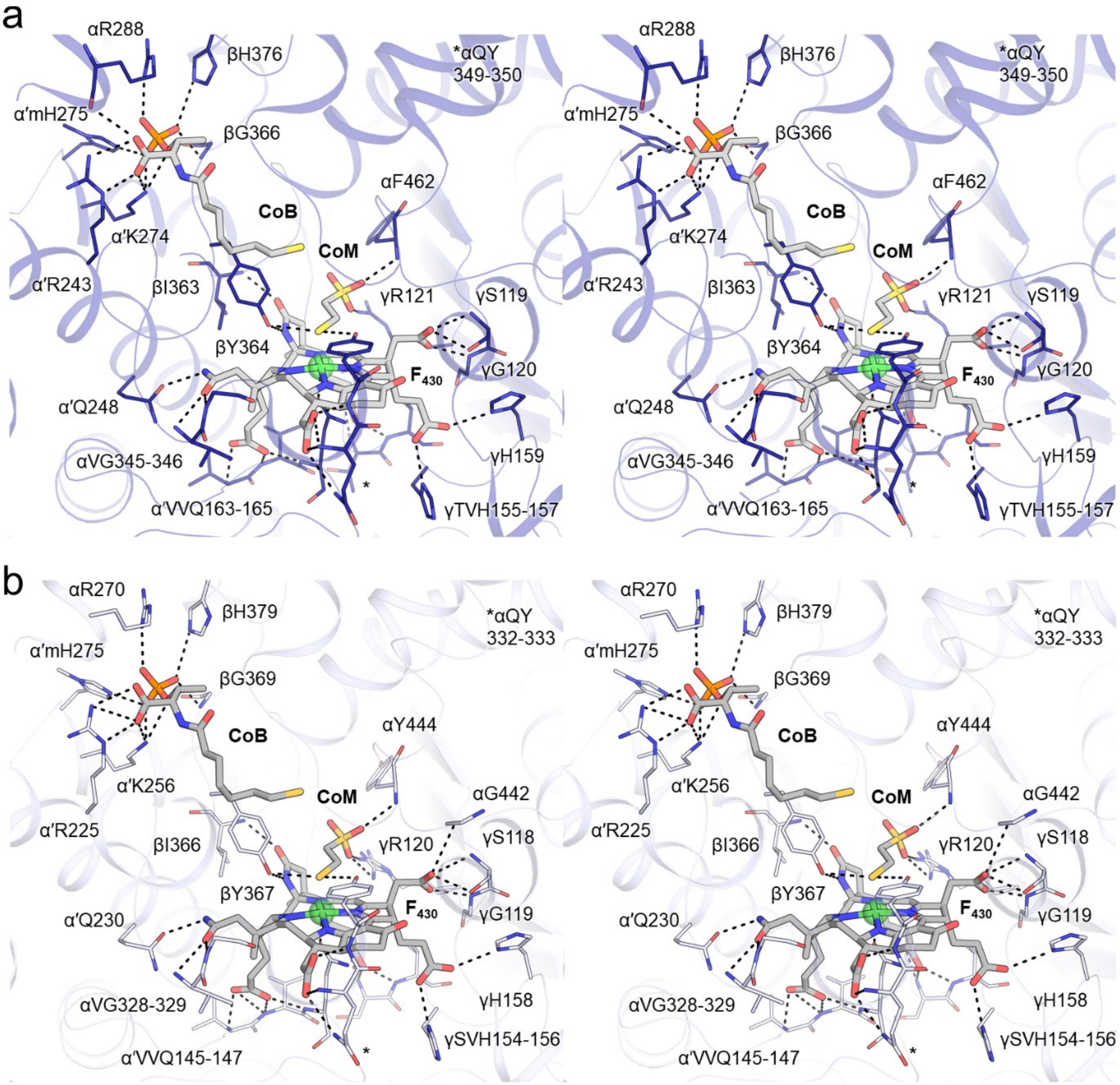

Supplementary Figure S10. Continued on the next page.

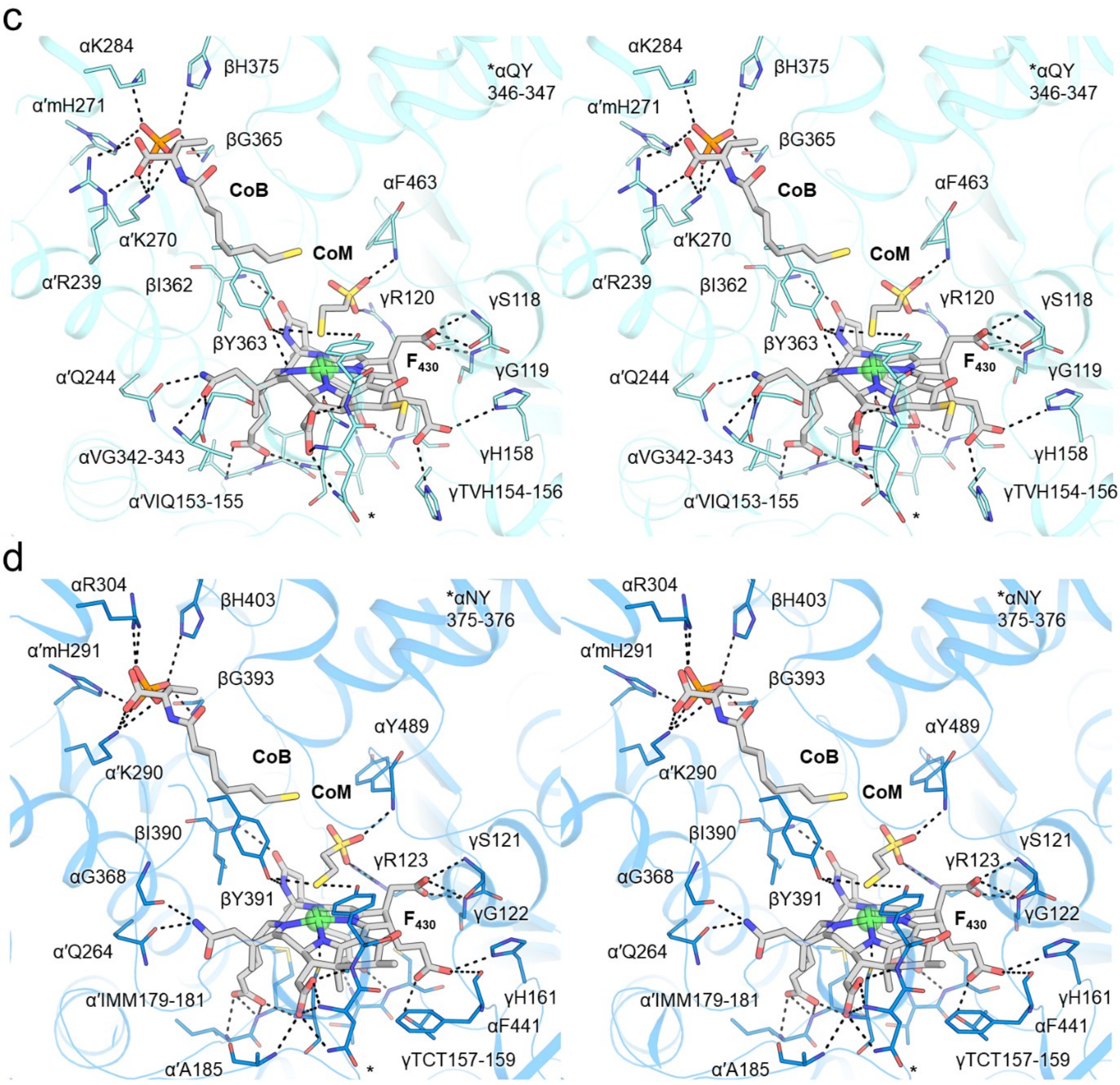

Supplementary Figure S10. Active site of MCR homologs. Stereo-view of the MCR active site in (**a**) *M. shengliensis* (7NKG), (**b**) MCR isoform I from *M. marburgensis* (5A0Y), (**c**) ANME-1 (3SQG), and (**d**) '*Ca*. E. thermophilum' (7B1S). In ANME-1 and *Ca*. E. thermophilum the presented F_{430} contains a methylthio and dimethyl addition, respectively. The main chains are shown as cartoons, and ligands are shown as sticks coloured according to the atom with red for O, blue for N, orange for P, green for Ni, and yellow for S. Coordinating residues are shown as lines, non-interacting mainchains were omitted. Polar contacts are depicted as black dashes.

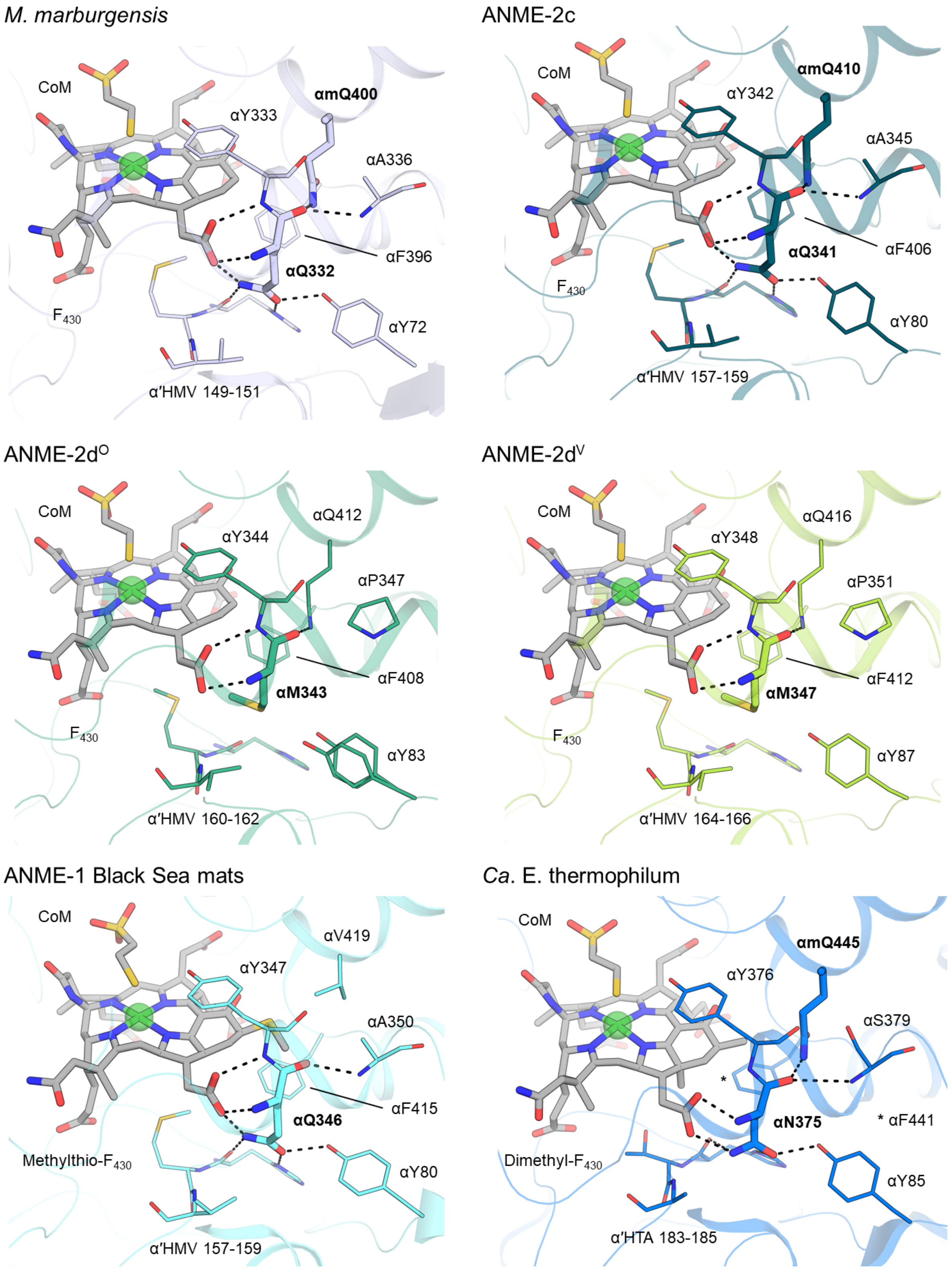

Supplementary Figure S11. Impact of the glutamine to methionine substitution in the vicinity of the F_{430}. MCR models are shown as a transparent cartoon, and the Gln/Met of interest and the PTM, cofactor F_{430} and CoM are highlighted in sticks. CoB and the heterodisulfide are not shown for clarity. Residues in the close environment of the Gln position 332 and equivalent in homologues are depicted in lines, with polar contacts as grey dashes. Atoms are coloured as follows: red for O, blue for N, orange for P, green for Ni, and yellow for S.

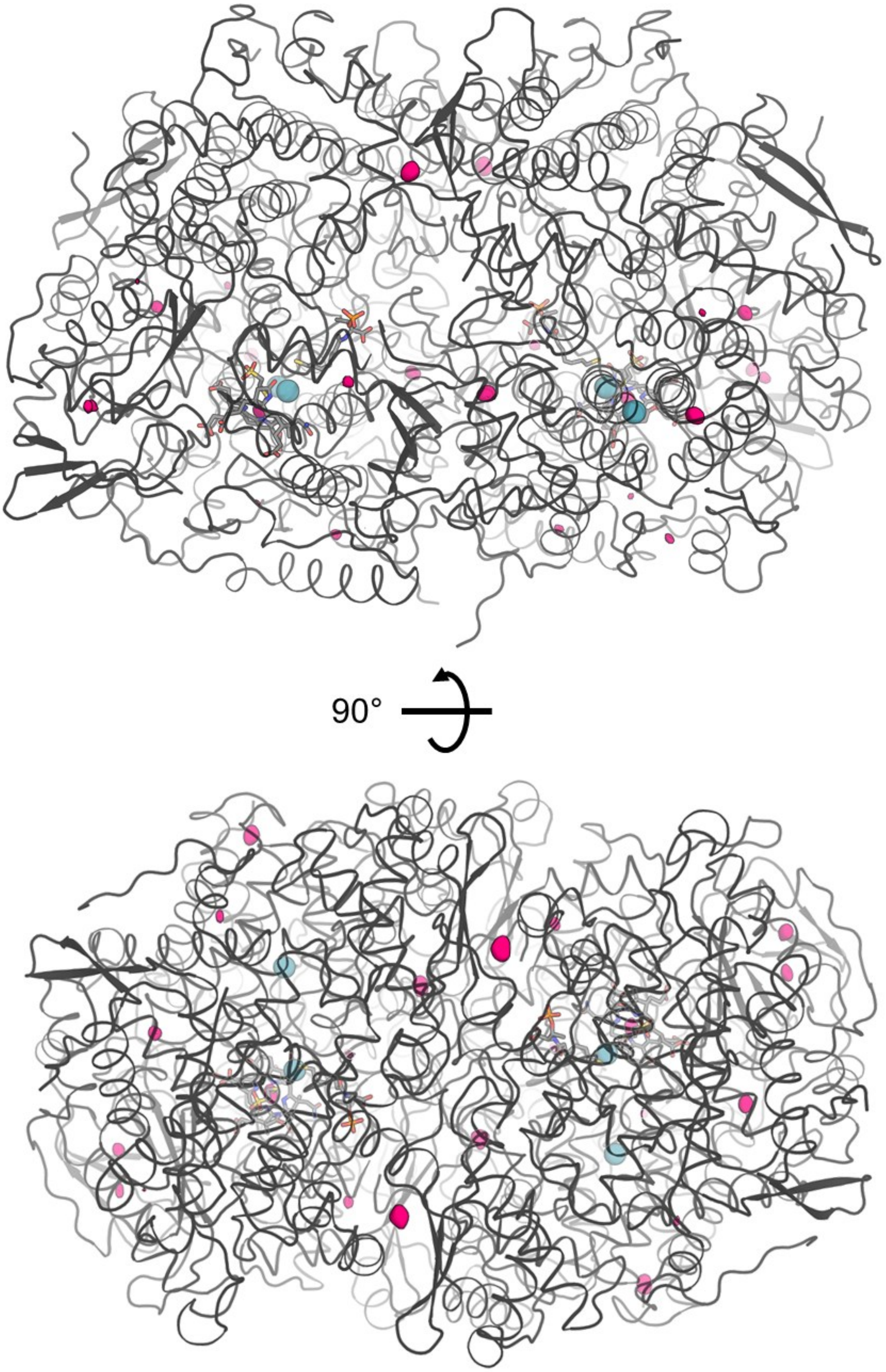

Supplementary Figure S12. Internal cavities of MCRs. Anomalous signal of Krypton is depicted by a pink surface contoured to 5 σ. The proteins are shown in cartoons with Xenon positions detected in the gassed structure of '*Ca*. E. thermophilum' (extracted from PDB 7B2C) shown in teal spheres. Coenzymes and F_{430} cofactors are shown as sticks.

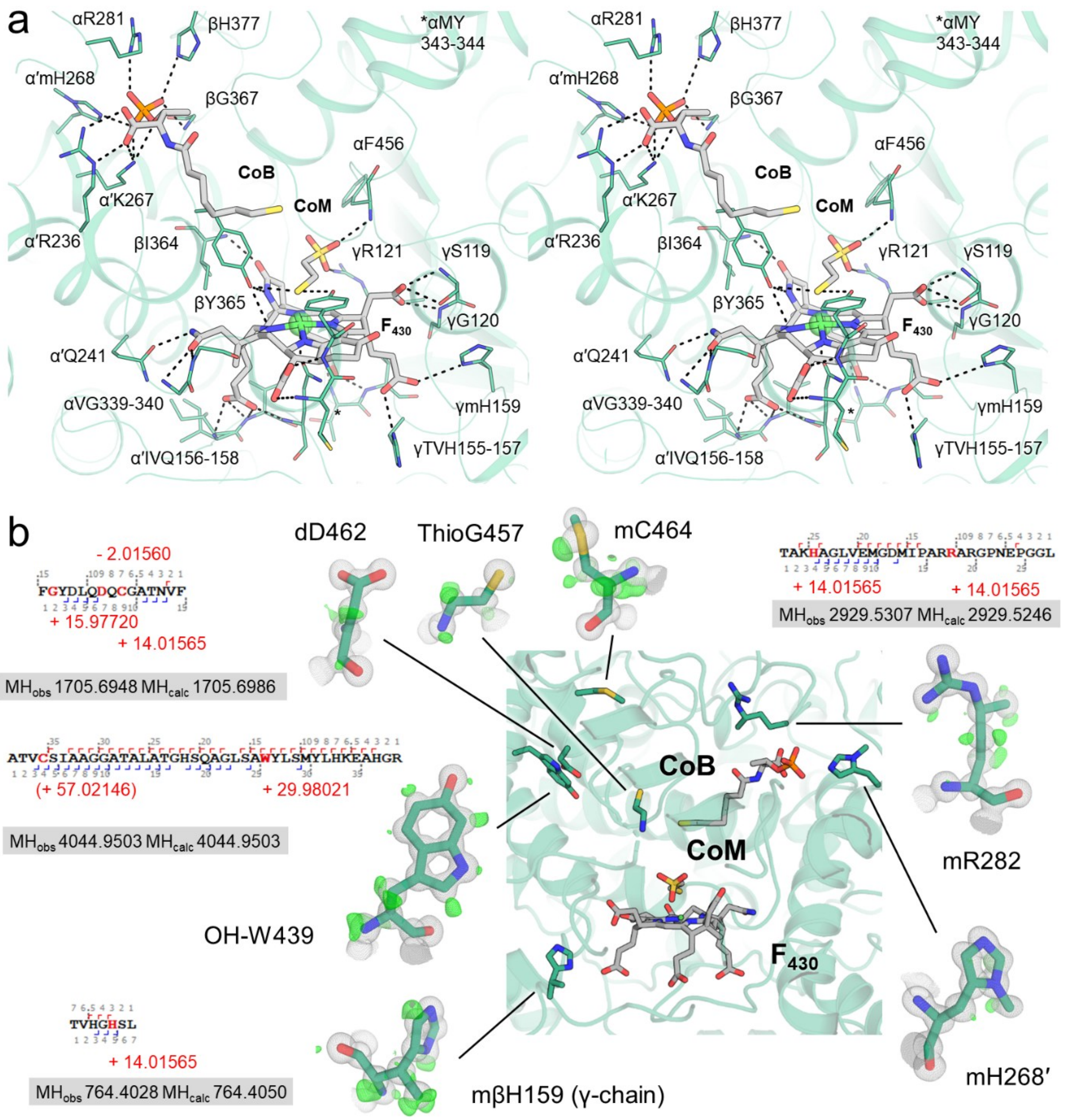

Supplementary Figure S13. Active site and PTMs in MCR from ANME-2d^{O}. (**a**) Stereo-view of the MCR active site in ANME-2d^{O}. Interacting residues are shown as lines, and non-interacting main chains were omitted. Interacting residues are labelled as follows: chain-one letter amino acid code-residue number. Polar contacts are in black dashes. (**b**) PTMs in the active site of ANME-2d^{O} with the same representation as in Fig. 4. The $2F_o - F_c$ map (grey mesh) is contoured at 2 σ, and the $F_o - F_c$ map (green mesh) is contoured at 3 σ. A representative peptide sequence with fragment ions (hooks) is shown for each PTM. Mass shifts are highlighted in red and shown in brackets if artefactual. The observed (MH_{obs}) and calculated (MH_{calc}) monoisotopic mass are shown in a box below. 6-hydroxytryptophan was treated with chymotrypsin, all others with trypsin. The hydroxytryptophan could not be found reliably with Byonic (+29.98021 instead of +15.99 Da) because this software tends to have a fanciful interpretation and the hydroxytryptophan in turn receives artificial modifications. However, its detection of H was very concise using Proteomediscoverer (Sequest) with a detected peptide "ATVCSIAAGGATALATGHSQAGLSAWYLSMYLHKEAHGR" MH_{obs} 4046.95783 Da and MH_{calc} 4046.960023 Da.

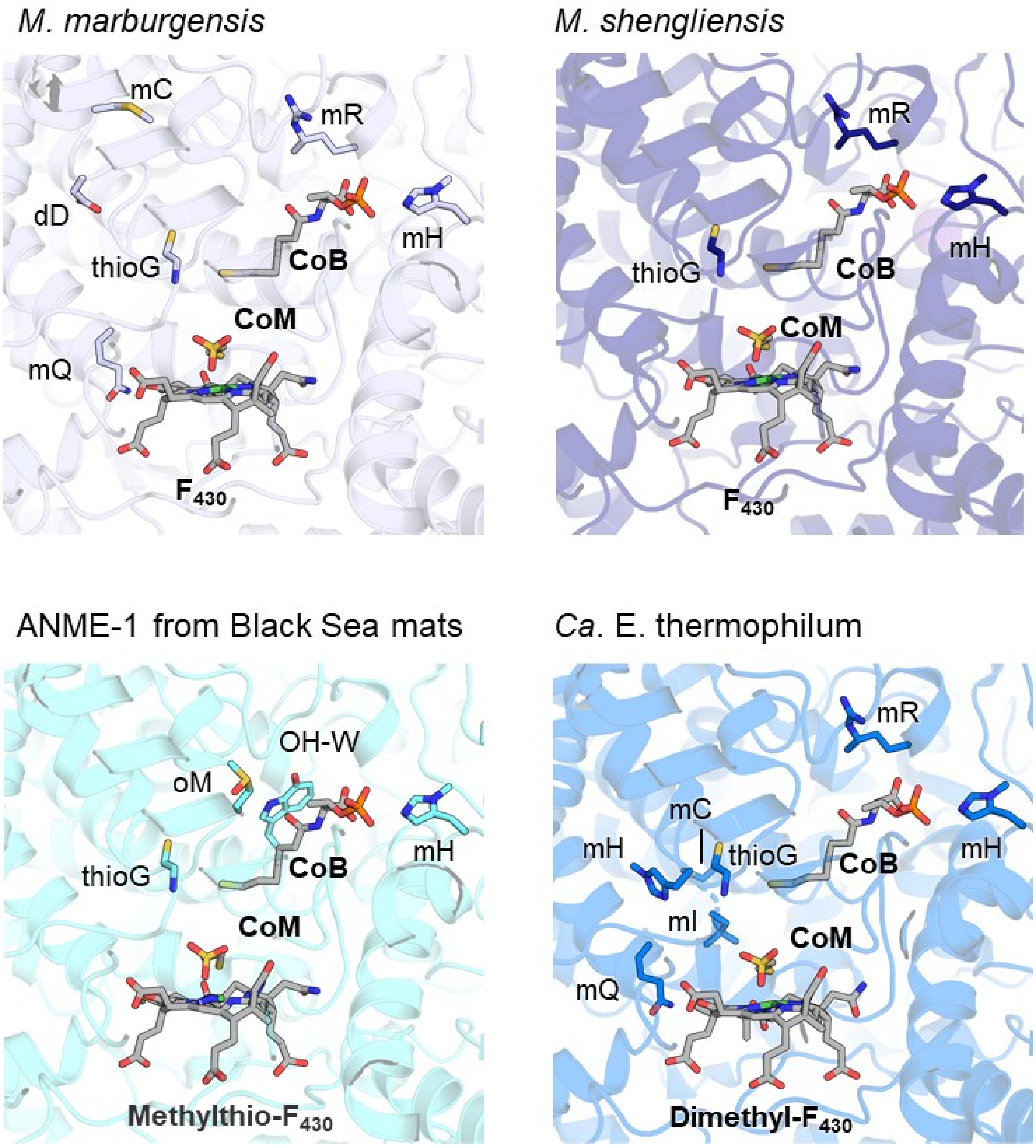

Supplementary Figure S14. PTMs in MCR homologs. The main chain is shown as a cartoon, with ligands and PTMs depicted as sticks coloured according to atom (red for O, blue for N, orange for P, green for Ni, and yellow for S). Residues are labelled according to the 1-letter ligand codes, with mH, thioG, mR, mQ, mC, dD, oM, mI, and OH-W standing for N^1-methylhistidine, thioglycine, 5(*S*)-methylarginine, 2(*S*)-methylglutamine, S-methylcysteine, didehydroaspartate, S-oxymethionine, 3-methylisoleucine, and 7-hydroxy-L-tryptophan, respectively. In *Ca*. E. thermophilum, the second mH close to mI is a N^2-methylhistidine.

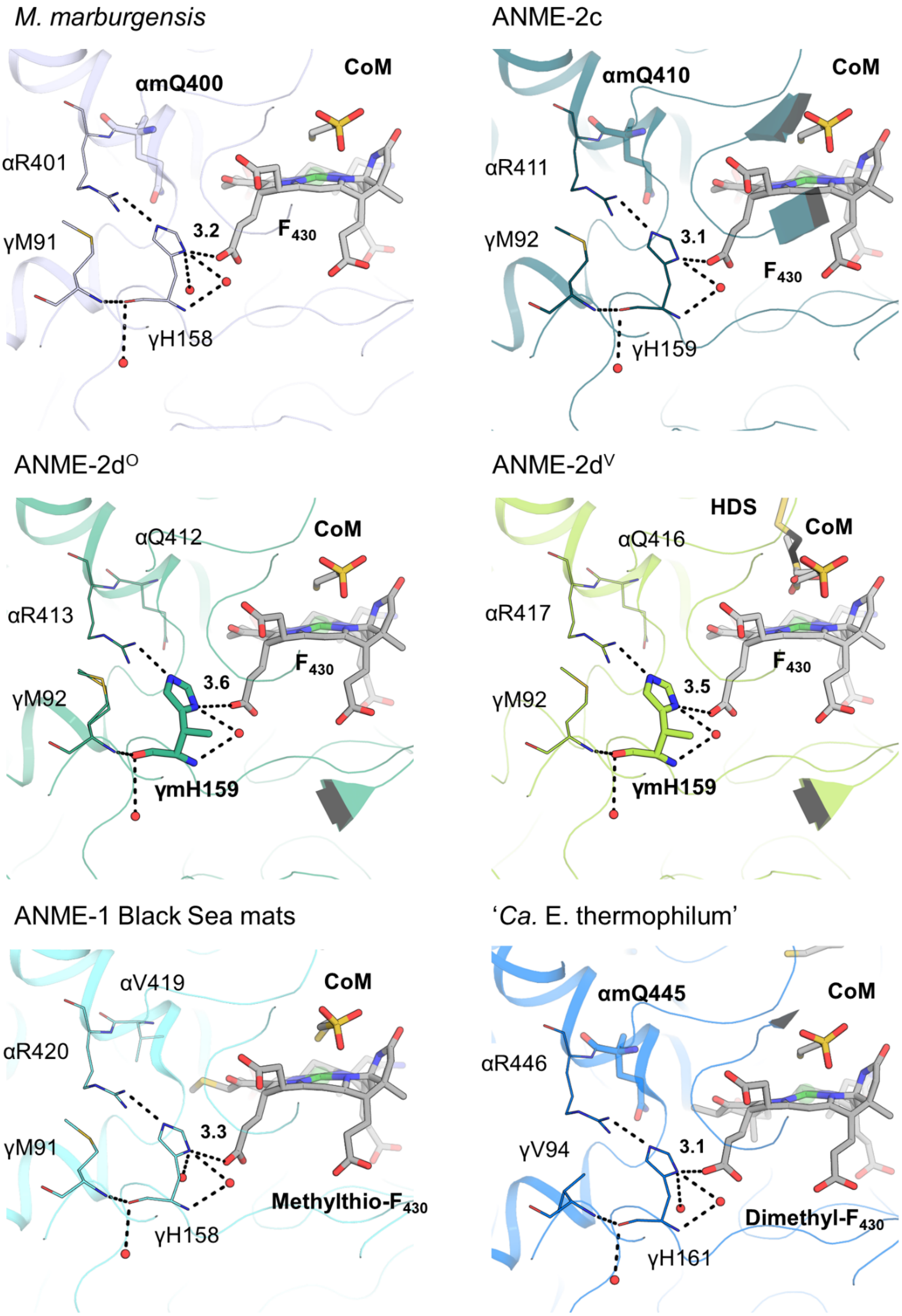

Supplementary Figure S15. Continued on the next page.

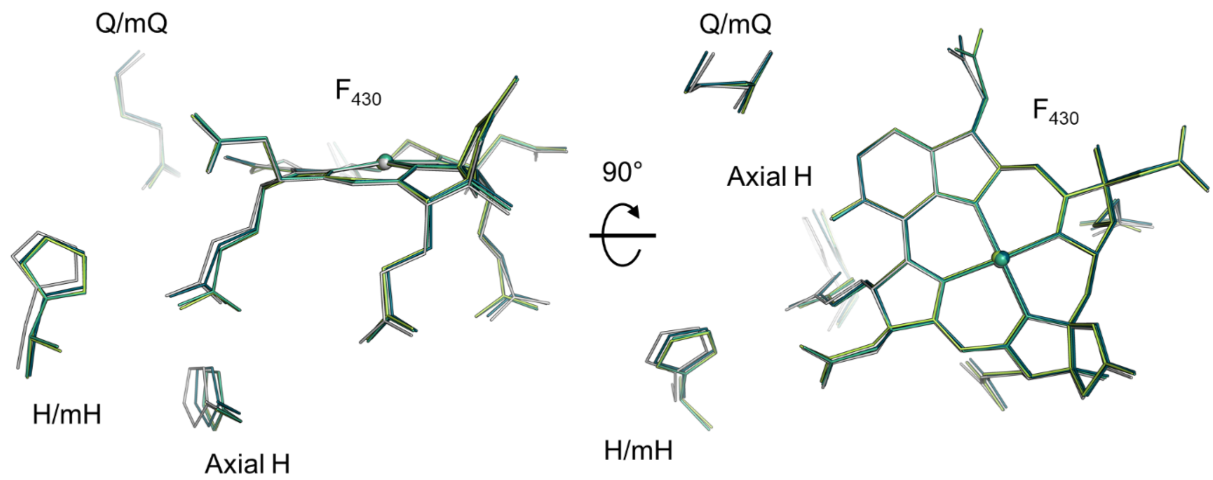

Supplementary Figure S15. Environment around the 3(*S*)-methylhistidine. First page: The 3(*S*)-methylhistidine or the equivalent unmodified histidine is shown for the different MCR models. The main chain is depicted as a cartoon, and ligands are shown as sticks coloured as Supplementary Fig. S14. PTMs are shown as sticks and near residues as lines. Polar contacts of histidine/3(*S*)-methylhistidine are depicted as grey dashes. Interacting waters are shown as spheres. Second page: superposition of α, β, and γ chains from the MCR of *M. marburgensis* (light grey) and MCR ANME-2 (same colour code as Supplementary Fig. S14) to highlight position discrepancies in the F_{430}. The positions of mQ/Q (e.g., αmQ410 in ANME-2c), mH/H (e.g., γmH159 in MCR ANME-2d^{O} and ANME-2d^{V}), and the axial histidine engaging a salt bridge with the F_{430} propionate group (e.g., γmH157 in MCR ANME-2d^{O} and ANME-2d^{V}) are shown as sticks. Nickel is shown as a sphere.

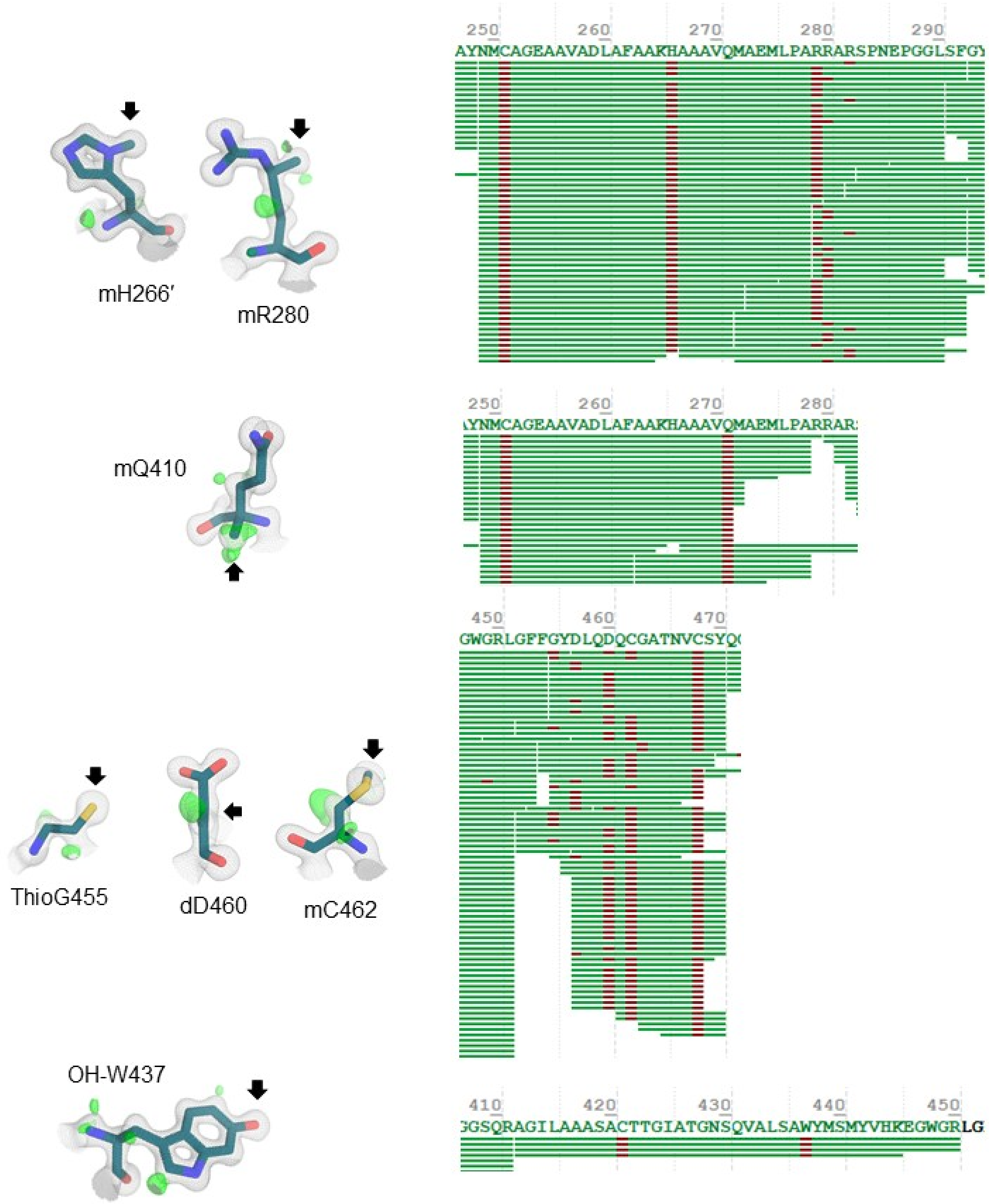

Supplementary Figure S16. PTMs in ANME-2c MCR detected by LC-MS analysis. From left to right: Post-translationally modified residue as sticks with the $2F_o{-}F_c$ (grey mesh) and $F_o{-}F_c$ map (green mesh) contoured at 2 and 3 σ, respectively. Black arrows highlight the modified position. An alignment of fragments is shown on the right with modified positions highlighted in red. 6-hydroxytryptophan was treated with chymotrypsin, all others with trypsin.

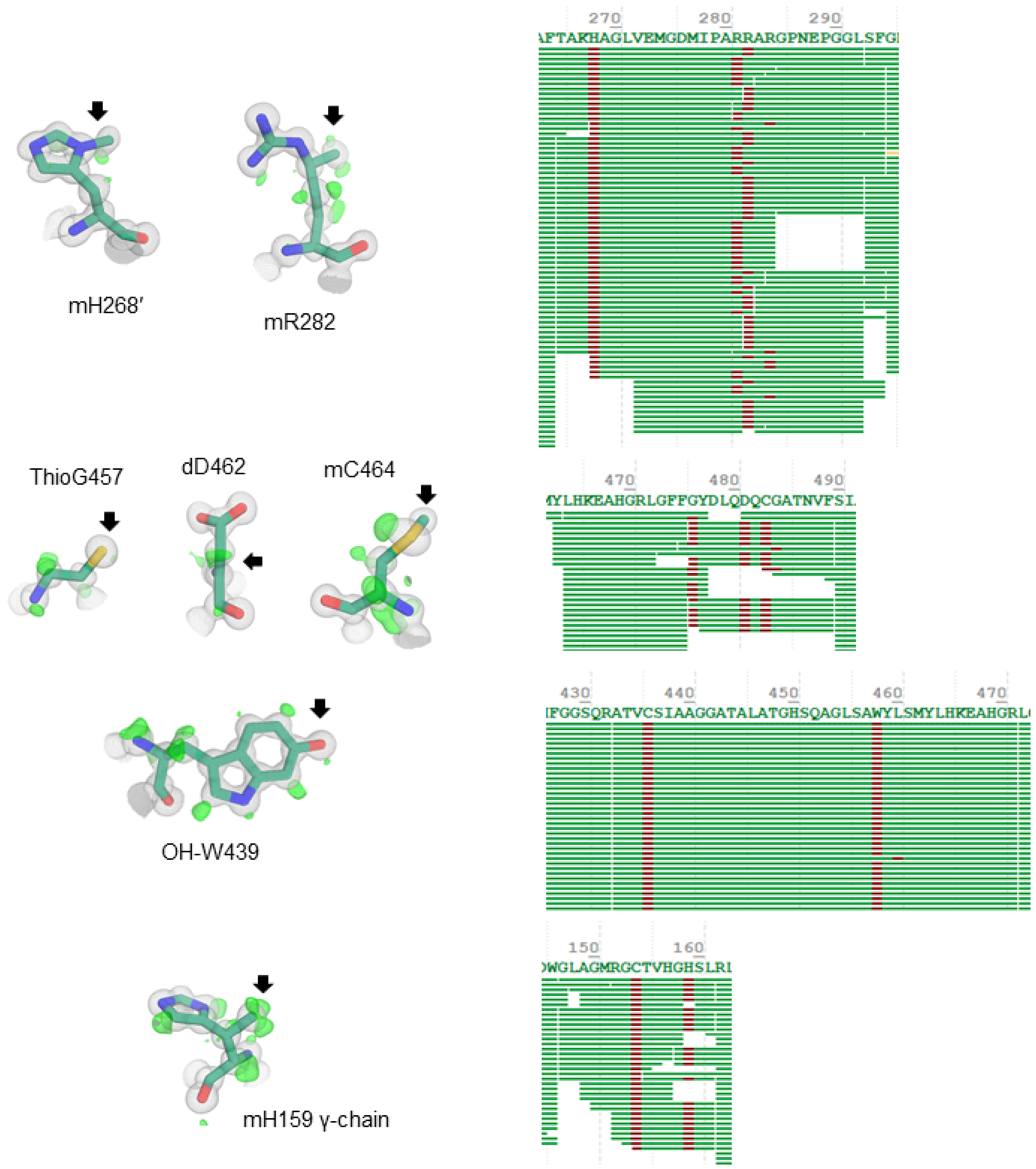

Supplementary Figure S17. PTMs in ANME-2d^{O} MCR detected by LC-MS analysis. From left to right: Post-translationally modified residue as sticks with the $2F_o$–F_c (grey mesh) and F_o–F_c map (green mesh) contoured at 2 and 3 σ, respectively. Black arrows highlight the modified position. An alignment of fragments is shown on the right with modified positions highlighted in red.

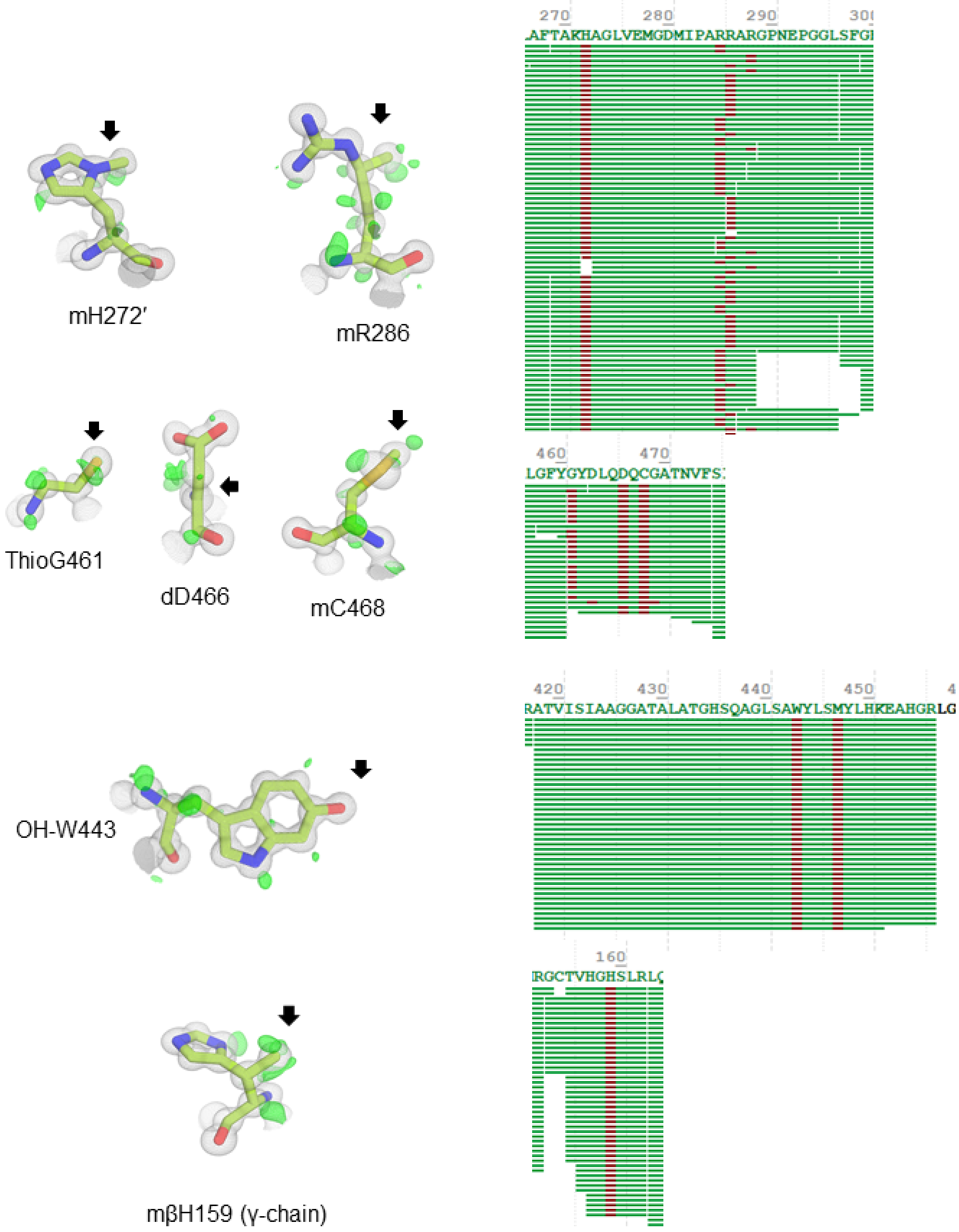

Supplementary Figure S18. PTMs in ANME-2d^{V} MCR detected by LC-MS analysis. From left to right: Post-translationally modified residue as sticks with the $2F_o-F_c$ (grey mesh) and F_o-F_c map (green mesh) contoured at 2 and 3 σ, respectively. An alignment of fragments is shown on the right with modified positions highlighted in red.

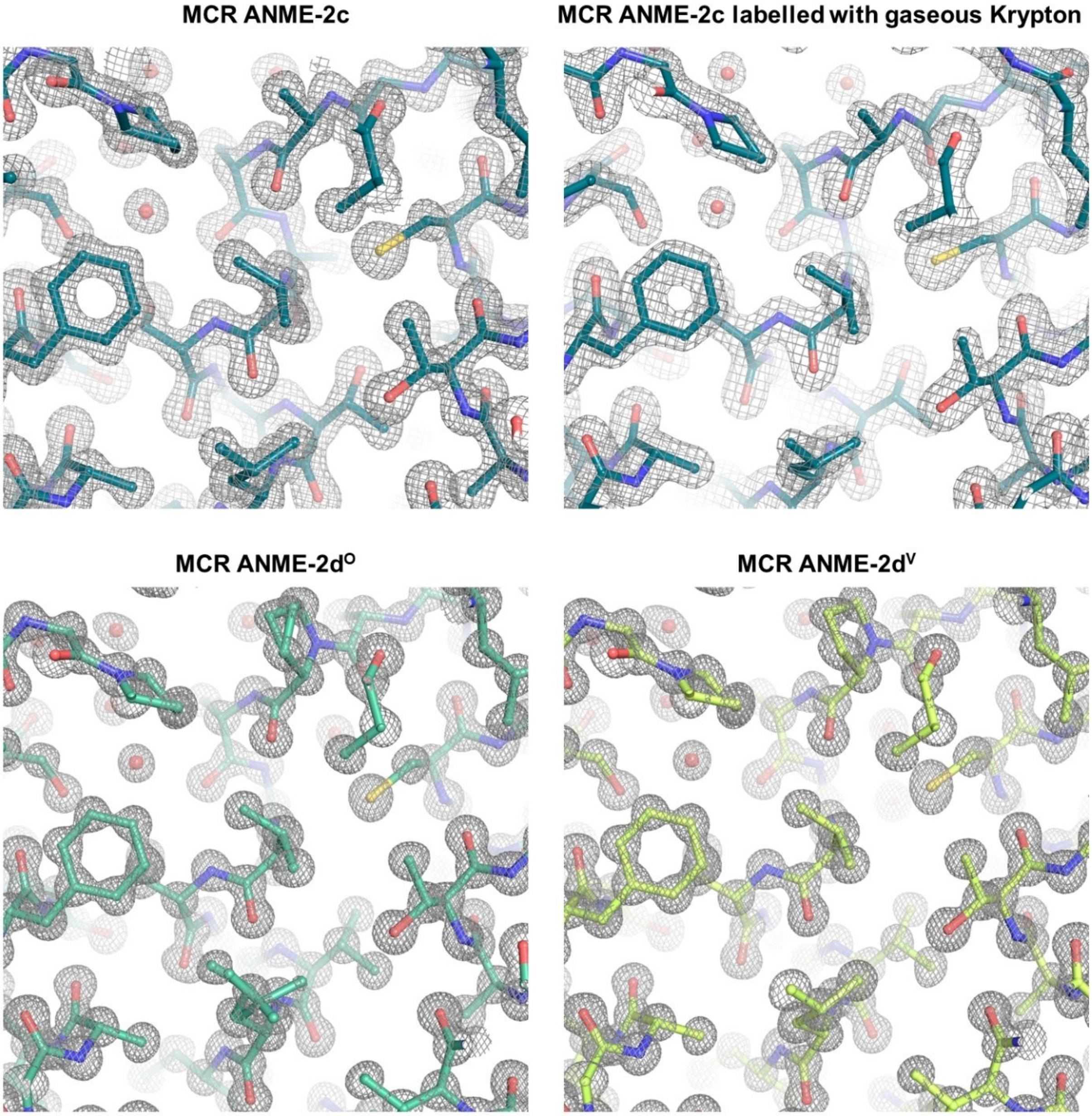

Supplementary Figure S19. Local electron density in the crystal structures obtained in this work. Portion of an electron density map for the four crystal structures with residues displayed as balls and sticks and coloured as in Figure S11. The $2F_o$−F_c maps are all contoured to 2 σ.

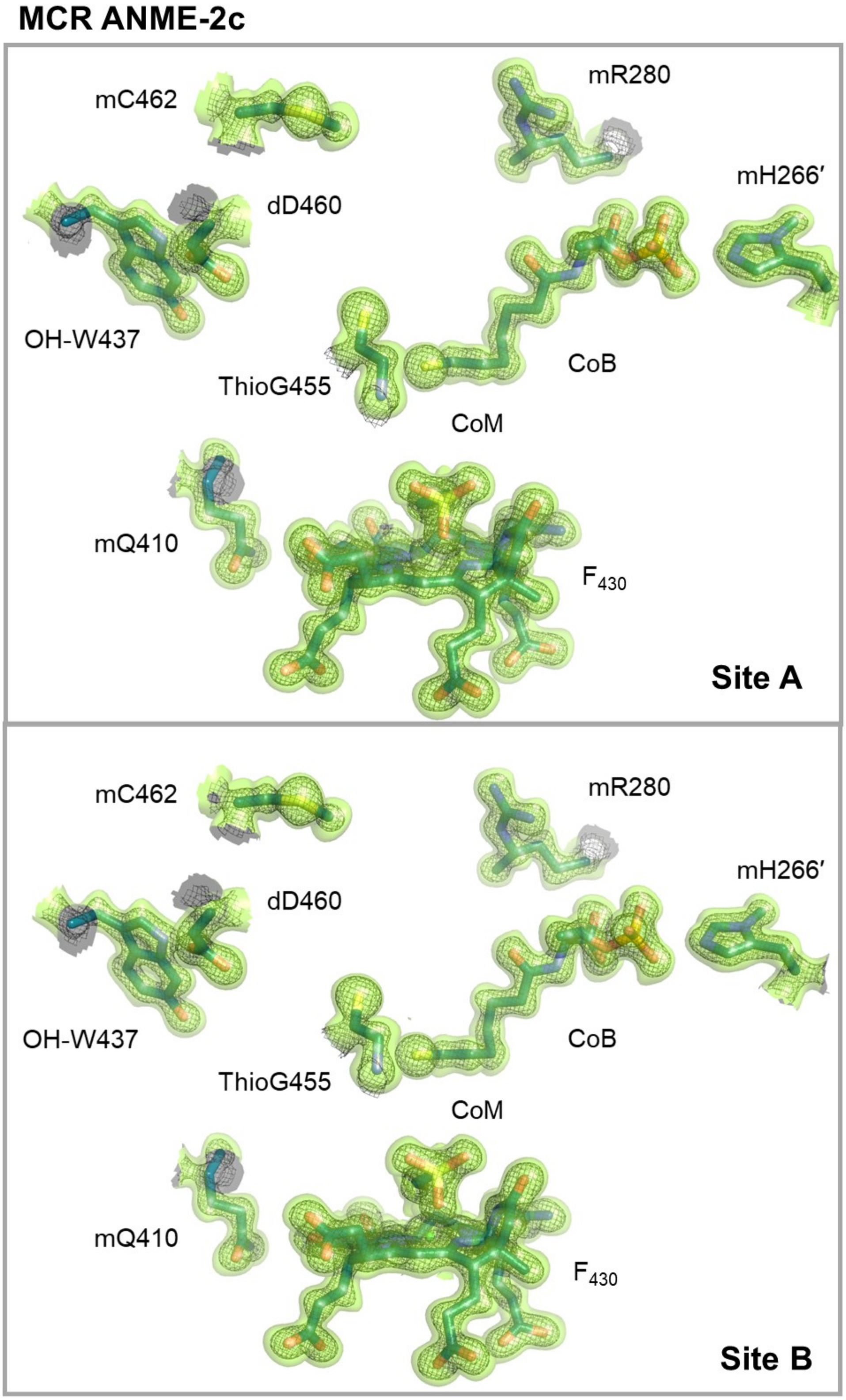

Supplementary Figure S20. Omit map for ligands in the MCR ANME-2c structure. Ligands and PTMs are displayed as balls and sticks and coloured as in Figure S11. $2F_o{-}F_c$ (contoured to 2 σ) and $F_o{-}F_c$ omit maps (contoured to 3 σ) are shown in black mesh and transparent green surface, respectively.

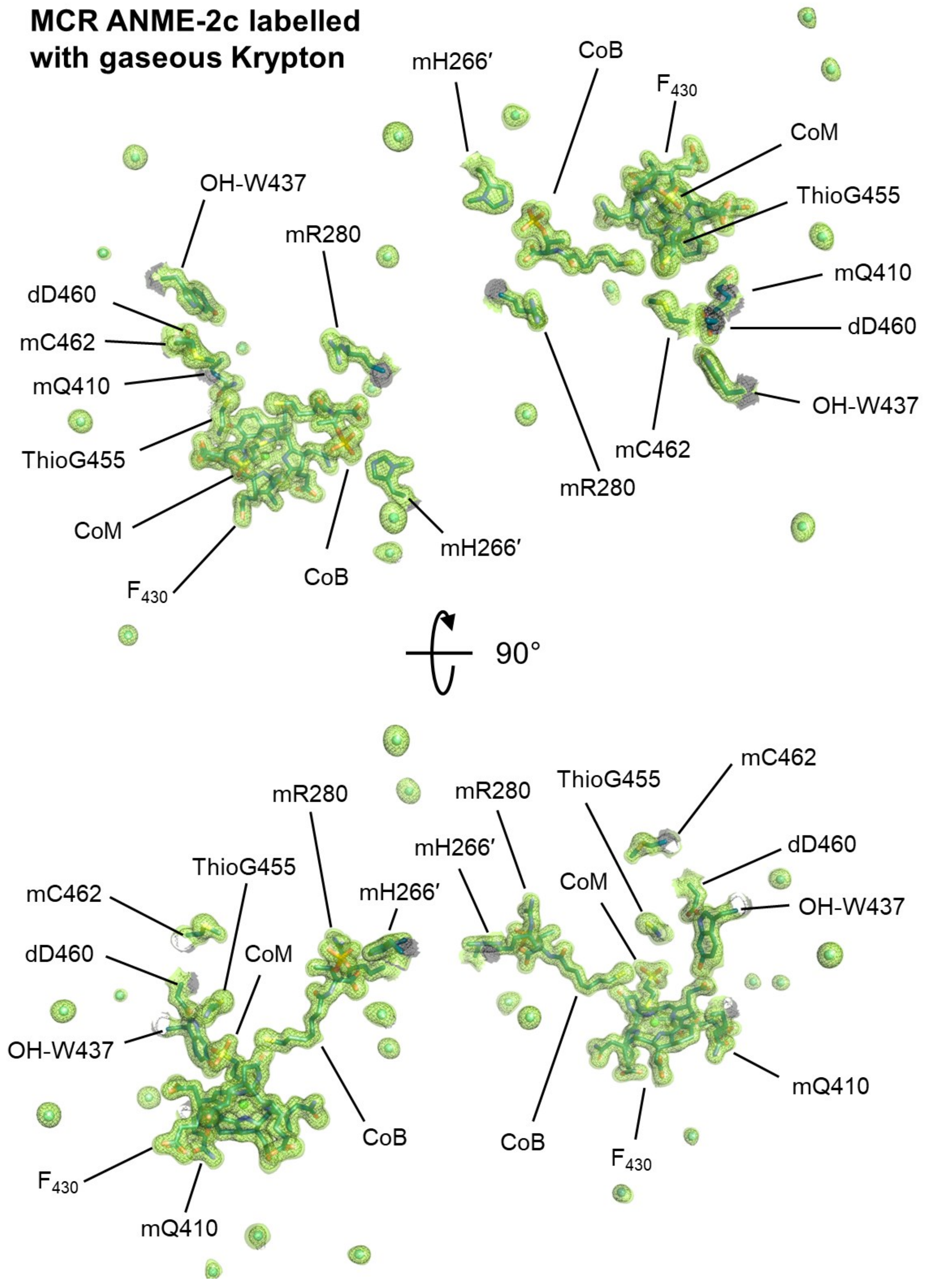

Supplementary Figure S21. Omit map for ligands in the MCR ANME-2c krypton-gassed structure. Ligands and PTMs are displayed as balls and sticks and coloured as in Figure S11. Green spheres correspond to modelled Krypton. $2F_o-F_c$ (contoured to 1 σ) and F_o-F_c omit maps (contoured to 3 σ) are shown in black mesh and transparent green surface, respectively. Both sites are presented with a 90° rotation.

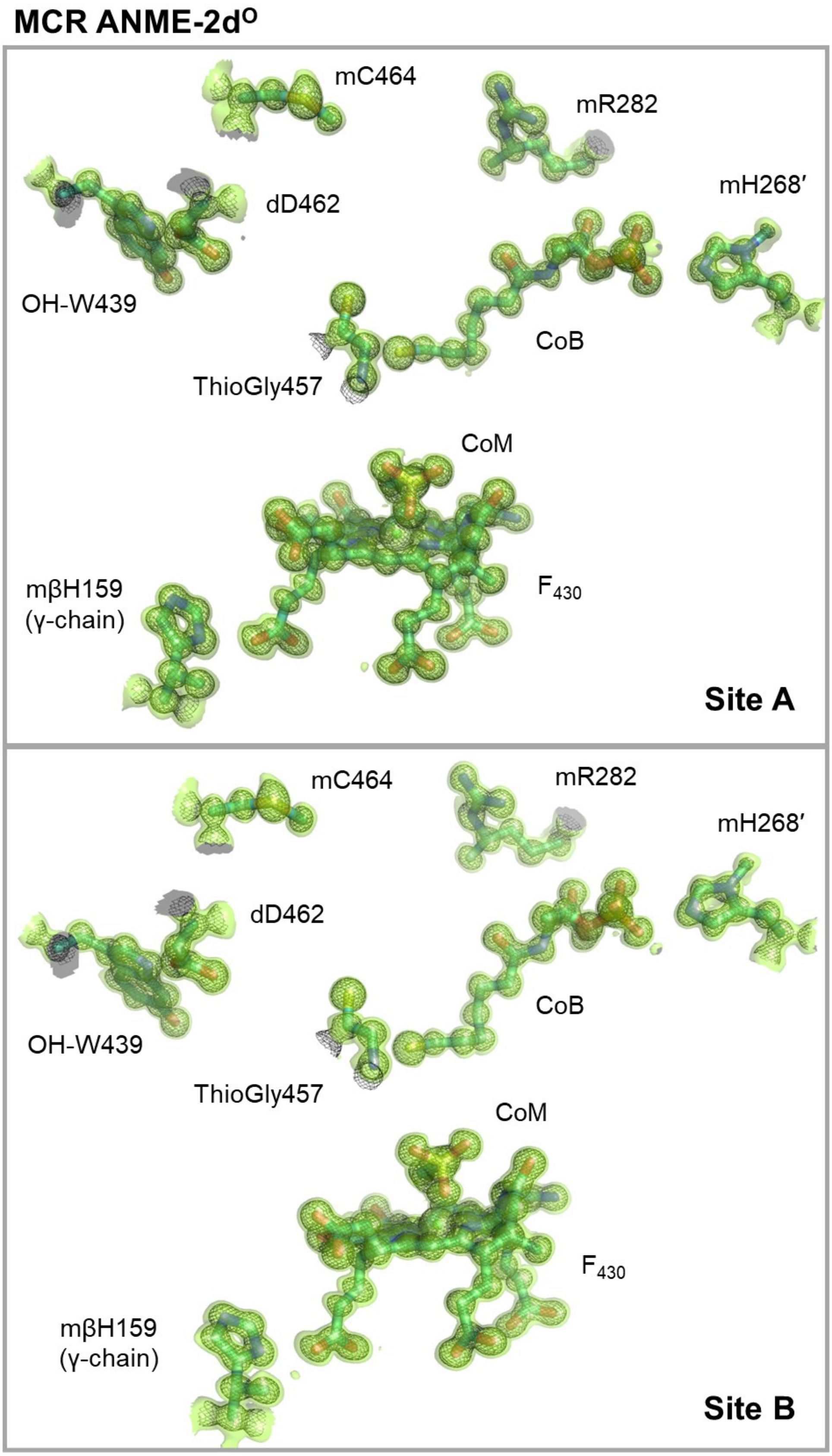

Supplementary Figure S22. Omit map for ligands in the MCR ANME-2d^{O} structure. Ligands and PTMs are displayed as balls and sticks and coloured as in Figure S11. $2F_o-F_c$ (contoured to 2 σ) and F_o-F_c omit maps (contoured to 3 σ) are shown in black mesh and transparent green surface, respectively.

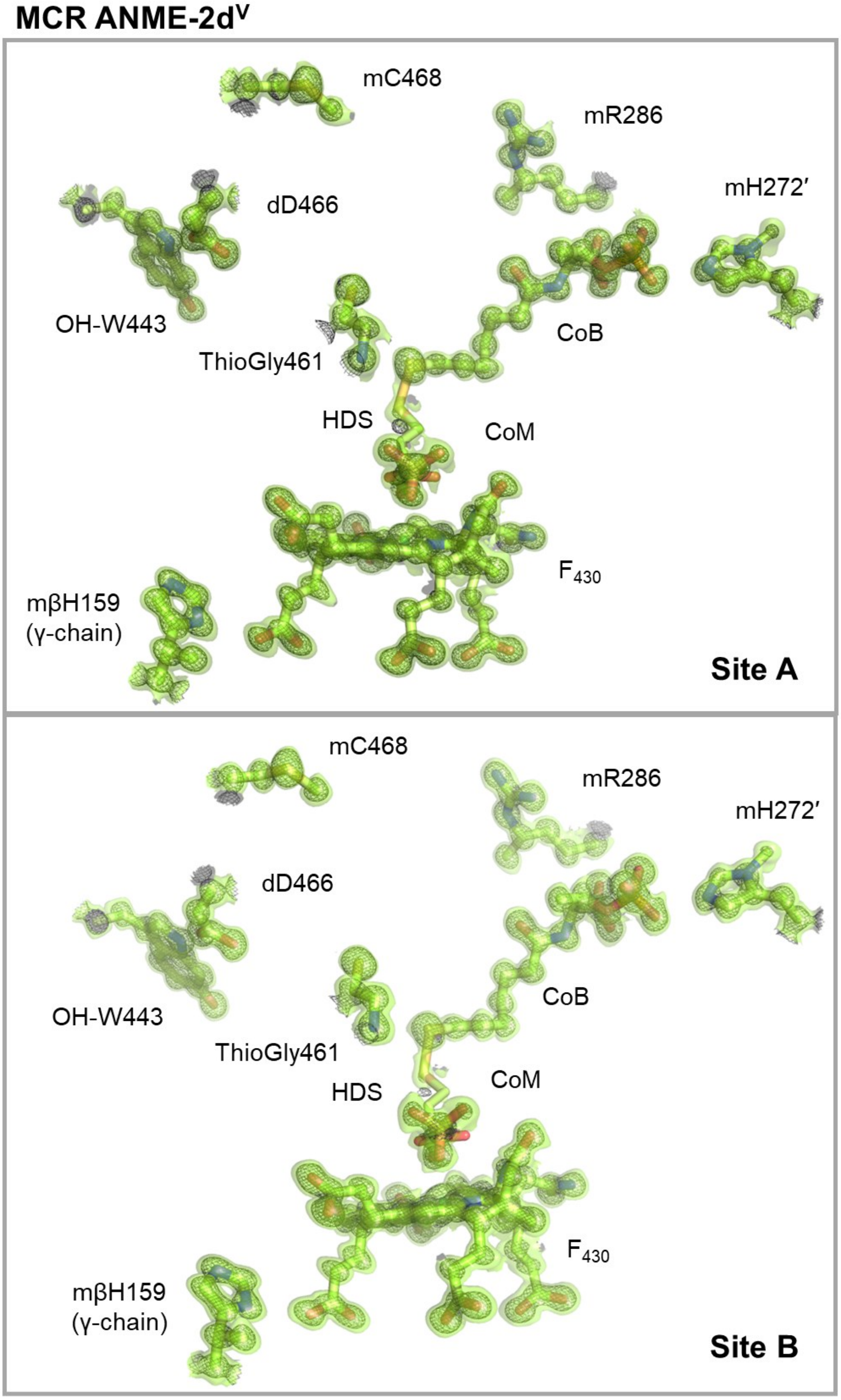

Supplementary Figure S23. Omit map for ligands in the MCR ANME-2d^{V} structure. Ligands and PTMs are displayed as balls and sticks and coloured as in Figure S11. $2F_o\text{−}F_c$ (contoured to 2 σ) and $F_o\text{−}F_c$ omit maps (contoured to 3 σ) are shown in black mesh and transparent green surface, respectively.

Supplementary references

1. Woodcroft BJ, Aroney STN, Zhao R, Cunningham M, Mitchell JAM, Blackall L, et al. SingleM and Sandpiper: Robust microbial taxonomic profiles from metagenomic data. BioRxiv. 2024.
2. Hahn CJ, Lemaire ON, Kahnt J, Engilberge S, Wegener G, Wagner T. Crystal structure of a key enzyme for anaerobic ethane activation. Science. 2021;373(6550):118-21.
3. Robert X, Gouet P. Deciphering key features in protein structures with the new ENDscript server. Nucleic Acids Res. 2014;42(W1):W320-W4.
